# The constitutive differential transcriptome of a brain circuit for vocal learning

**DOI:** 10.1186/s12864-018-4578-0

**Published:** 2018-04-03

**Authors:** Peter V. Lovell, Nicole A. Huizinga, Samantha R. Friedrich, Morgan Wirthlin, Claudio V. Mello

**Affiliations:** 1 Department of Behavioral Neuroscience, Oregon Health and Sciences University, 3181 Sam Jackson Park Rd L470, Portland, OR USA; 2 Current affiliation: Computational Biology, Carnegie Mellon University, Pittsburgh, PA USA

**Keywords:** molecular, speech and language, birdsong, cDNA microarray, oligo array, gene expression, brain, vocal learning

## Abstract

**Background:**

The ability to imitate the vocalizations of other organisms, a trait known as vocal learning, is shared by only a few organisms, including humans, where it subserves the acquisition of speech and language, and 3 groups of birds. In songbirds, vocal learning requires the coordinated activity of a set of specialized brain nuclei referred to as the song control system. Recent efforts have revealed some of the genes that are expressed in these vocal nuclei, however a thorough characterization of the transcriptional specializations of this system is still missing. We conducted a rigorous and comprehensive analysis of microarrays, and conducted a separate analysis of 380 genes by *in situ* hybridizations in order to identify molecular specializations of the major nuclei of the song system of zebra finches (*Taeniopygia guttata*), a songbird species.

**Results:**

Our efforts identified more than 3300 genes that are differentially regulated in one or more vocal nuclei of adult male birds compared to the adjacent brain regions. Bioinformatics analyses provided insights into the possible involvement of these genes in molecular pathways such as cellular morphogenesis, intrinsic cellular excitability, neurotransmission and neuromodulation, axonal guidance and cela-to-cell interactions, and cell survival, which are known to strongly influence the functional properties of the song system. Moreover, an in-depth analysis of specific gene families with known involvement in regulating the development and physiological properties of neuronal circuits provides further insights into possible modulators of the song system.

**Conclusion:**

Our study represents one of the most comprehensive molecular characterizations of a brain circuit that evolved to facilitate a learned behavior in a vertebrate. The data provide novel insights into possible molecular determinants of the functional properties of the song control circuitry. It also provides lists of compelling targets for pharmacological and genetic manipulations to elucidate the molecular regulation of song behavior and vocal learning.

**Electronic supplementary material:**

The online version of this article (10.1186/s12864-018-4578-0) contains supplementary material, which is available to authorized users.

## Background

Vocal learning, the ability to imitate the vocal sounds produced by other individuals, serves as the basis for spoken language acquisition in humans. It is a relatively rare trait that is thought to have evolved in a few other mammalian groups (e.g. cetaceans, bats) and in three orders of birds (hummingbirds, parrots, songbirds [1–4]). Most thoroughly studied in the zebra finch (*Taeniopygia guttata*), a representative songbird species, the acquisition, memorization, and production of learned birdsong requires the coordinated activity of a set of specialized brain nuclei that are collectively referred to as the song control system [1, 2, 5–7]. Decades of intensive research have revealed much about the organization, connectivity, and physiological properties of these nuclei, collectively referred to as the song control system, in the context of vocal learning and song production (see reviews in [6, 7]).

**Fig. 1:**
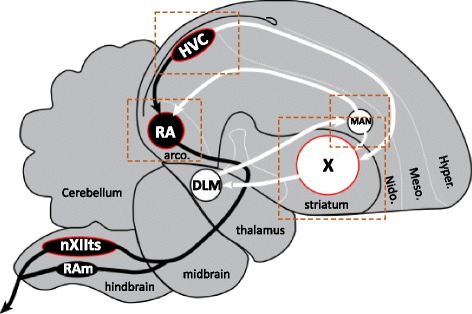
Major brain areas for vocal learning and singing in zebra finches. Diagram of the songbird brain in the parasagittal plane illustrating the relative positions and connections between the major nuclei of the song control system. Several related nuclei and connections have been removed for clarity. Song nuclei analyzed in the present study are outlined in red. The song system consists of the direct motor pathway (DMP) for song production (nuclei and projections in black), and an anterior forebrain pathway (AFP) for song learning and plasticity (nuclei and projections in white). Dotted rectangles indicate the approximate positions of the photomicrographs presented in the panels in Figs. 10, 11, 12, 13 and 14. Abbreviations: HVC, proper name; RA, robust nucleus of the arcopallium; LMAN, lateral magnocellular nucleus of the anterior nidopallium; nXIIts, tracheosyringeal portion of the hypoglossal nerve nucleus; RAm, nucleus retroambigualis; X, striatal Area X; Hyper., hyperpallium; Meso., mesopallium; Nido., nidopallium; Arco., arcopallium. For nomenclature details see [89]

The song control system is organized into two distinct but interconnected pathways, consisting of a direct vocal-motor pathway (DMP; whereas nXIIts markers were mostly Fig. 1; nuclei and projections in black), which is required for the production of song [8, 9], and an anterior forebrain pathway (AFP; Fig. 1; nuclei and projections in white), which is required for the acquisition of song [10–12], and adult vocal plasticity [13–16]. The DMP is comprised of pre-motor pallial (cortical-like) nucleus HVC, which sends axonal projections to the robust nucleus of the arcopallium (RA), the vocal-motor output nucleus of the telencephalon. RA, in turn, projects to several brainstem targets that comprise a vocal-respiratory network, most prominently the tracheosyringeal part of the hypoglossal nucleus (nXIIts), which innervates the syrinx, the avian vocal organ [8, 17]. While RA is considered analogous to vocal-motor projection neurons in layers 5/6 of human laryngeal motor cortex (LMC), its brainstem target (nXIIts) is analogous to mammalian nucleus ambiguous, a nucleus that innervates the larynx, the vocal organ in mammals. In adults, lesioning the nuclei of the DMP results in disrupted singing [8, 18], while microstimulation can evoke calls or song depending upon the brain level stimulated [19]. In contrast, the AFP is initiated by a projection from HVC to Area X, a specialized striato-pallidal nucleus. In turn, Area X projects to the medial part of the dorsolateral anterior thalamic nucleus (DLM), which projects to the lateral magnocellular nucleus of the anterior nidopallium (LMAN). Neurons in LMAN project both to Area X, forming a cortico-striatal-thalamo-cortico loop that is reminiscent of the loops that are important for sensorimotor integration and motor learning behavior in mammals [20, 21], and to RA, providing input into the DMP. Lesions to these nuclei during the song learning period prevent the acquisition of song by imitation of adults [12, 22]. Of note, vocal nuclei from both pathways above are absent in non-vocal learning birds (e.g. chicken, owl, woodpecker).

Importantly, individual vocal nuclei are embedded within distinct pallial or subpallial brain subdivisions, but also possess a number of unique features that differentiate them from their surrounds, including distinct cell types, unique patterns of connectivity, exquisite sensitivity to sex steroids, and specialized electrophysiological and synaptic properties, to cite some examples (see reviews in [6, 7]). Vocal nuclei thus represent distinct anatomical and functional specializations of their respective brain subdivisions: HVC and LMAN are specialized nuclei of the nidopallium, RA of the arcopallium, and Area X of the striatum (Fig. 1). Identifying genes that are differentially expressed between individual vocal nuclei and their surrounds provides an opportunity to identify programs of differential gene expression potentially linked to the unique properties of these vocal nuclei. Studies demonstrating the differential expression of sex steroid receptors and retinoid synthesis genes in several song nuclei provided early evidence that distinct transcriptional regulation likely occurs within song nuclei compared to their surrounds. Large scale gene microarray screenings and *in situ* hybridization studies in zebra finches and other songbird species like song sparrows, canaries, and starlings have provided further evidence that song nuclei possess unique programs of constitutive [23–29], seasonally changing [30, 31], and singing-induced gene expression [27, 28, 32–35], the varying results across studies and species likely reflecting the specific roles that song nuclei play in different aspects of vocal behavior and learning (see also [36]). While laying the groundwork for identifying previously unknown properties of the song system, such studies have also helped build new hypotheses about how the unique properties of the song system are regulated at the molecular level. They have also helped uncover remarkable evidence of convergent molecular specializations of cortical and striatal vocal areas between songbirds and humans [23]. Despite these advances, a comprehensive and comparative analysis of the constitutive molecular specializations of the major song nuclei of songbirds has not yet been performed.

To address this gap in knowledge we have conducted a thorough transcriptomics analysis of the major zebra finch vocal nuclei that make up the DMP, namely HVC, RA, and nXIIts, as well as of the primary basal ganglia nucleus of the AFP, namely Area X (Fig. 1; the nuclei analyzed are outlined in red). Our goal was to identify constitutive markers of zebra finch song nuclei based on contrasting expression with adjacent brain areas in adult males. This was accomplished by analyzing publicly available microarray data previously used to identify singing regulated genes [33, 35], convergent specializations of analogous vocal nuclei in birds and humans [23], and molecular specializations of HVC [24]. In addition, we analyzed *in situ* hybridization data for 380 genes, which not only greatly expanded the analysis, but also afforded us the opportunity to assess expression in nuclei not analyzed by the microarrays. Importantly, our study differs from previous studies in that we focused on identifying genes that are constitutively expressed in the song system of quiet, non-singing birds, thus excluding the confound of singing-induced expression. We also conducted rigorous and extensive annotation efforts for the microarrays, and used *in situ* hybridization patterns to independently establish high-confidence cut-off values for differential expression in each nucleus and analyze the expression in nuclei not analyzed by microarrays. We also performed an in-depth analysis of specific gene families of relevance to the development and physiology of the song system, including examination of *in situ* hybridization patterns for many highly relevant genes that were not sampled on the microarrays. Together, our efforts have identified over 2600 genes that are differentially regulated in one or in some cases several nuclei within the song system. Bioinformatics analyses provide substantial new insights into molecular pathways that guide cellular morphogenesis, neurogenesis and cell survival, steroidogenesis, intrinsic excitability, neurotransmission, neuronal connectivity, and steroid and retinoid sensitivity. Finally, our study provides an extensive list of genes that are compelling candidate targets for genetic or pharmacological manipulations aimed at further exploring the functional role of differential genes in the song system.

## Methods

### Analysis of cDNA and oligo microarray datasets

Our study consisted of 4 differential microarray screening experiments aimed at identifying molecular markers of HVC versus shelf (the area immediately ventral to HVC), RA versus ventrolateral arcopallium (VLA), striatal Area X versus ventral striato-pallidum (VSP), and nXIIts versus the supra-spinal medullary nucleus (SSP). In each case, samples were obtained from quiet, non-singing birds, which allowed us to assess the constitutive transcriptome. For song nucleus HVC, we analyzed a set of 12 spotted glass cDNA microarrays (ESTIMA:Song collection “20 k” cDNA microarrays, as detailed in [37]) previously used to screen for differentially expressed mRNAs in laser capture microdissected samples from HVC and the adjacent nidopallial shelf (*n* = 6 per sample; for details on sample preparation, mRNA isolation, probe amplification, labeling and hybridization, see Lovell et al. [24], Fig. 1). In the present study, we reanalyzed the datasets deposited for the HVC/Shelf study that are publically available from the Gene Expression Omnibus (GEO; GSE36270). For our reanalysis, we used a more robust data normalization and statistical analysis based on the R statistical package from BioConductor (http://www.bioconductor.org/). Briefly, the limma (Linear Models for Microarray analysis) R package [38] was used to read in the median foreground and median background fluorescence intensities. Background correction was performed using the “half” method, which subtracted the local background estimates from the foreground values, and then set any zero or negative values to 0.5 to avoid losing data. Within-array normalization was done using the “printtiploess” method to remove the dye intensity biases within each array. The resulting intensity values are in the form of M-values, which are log2(Cy5 / Cy3). However, all the experiments were done as a “common reference” design, where each sample was hybridized against the same common reference sample to facilitate comparisons across experiments. Dye-balancing was also performed, so that in half the arrays the reference was in the Cy5 channel in half the reference was in the Cy3 channel. Therefore, instead of M-values, we transformed the intensities so they were all log2(sample/reference). Between-group comparisons were calculated using the limma package in Bioconductor which uses a modified t-test to calculate *p*-values using an empirical Bayesian method to moderate the standard errors of the estimated log-fold changes [39]. Limma uses variance information from all the spots on the array to arrive at an estimate of per spot variance used in the t-tests. p-values were adjusted for multiple comparisons with the program’s q-value [40], which allows for selecting statistically significant genes while controlling the estimated “false discovery rate”.

For song nuclei RA, nXIIts, and Area X we performed statistical analyses of pre-normalized data sets that are publically available from NCBI Gene Expression Omnibus (GEO). Specifically, we analyzed sample sets that were previously hybridized to the Agilent Technologies Duke *Taeniopygia guttata* 45 K oligo array (for analysis of RA and nXIIts) or the 20 K Agilent-019785 Custom Zebra Finch Microarray (for the analysis of Area X). Complete descriptions of brain tissue sampling, mRNA isolation, oligo array hybridizations, gene expression normalization, and partial outcomes are available for nXIIts [35], RA [23], and Area X [32]. For our present analysis of these oligo arrays we used the GEOquery and limma R packages (NCBI Gene Expression Omnibus; [41] with the Benjamini & Hochberg false discovery rate method to identify differentially expressed oligos from the user-supplied normalized datasets. For RA, we analyzed 3 pairs of zebra finch samples from project GSE28395 (Avian arcopallium; [35] and performed ANOVA group comparisons between normalized expression values from RA (GSM701893, GSM701894, and GSM701895) and the ventrolateral arcopallium (VLA; GSM701896, and GSM701897, GSM7011898). For nXIIts, we analyzed 3 pairs of zebra finch samples from project GSE33667 (Avian 12th motor neuron; [23]) and performed ANOVA group comparisons between normalized expression values from nXIIts (GSM832502, GSM832503, and GSM832505) to those from the non-vocal supra-spinal medullary motor nucleus (SSP; GSM832501, and GSM832504, GSM832506). For Area X, we analyzed a subset of 9 quiet, non-singing control zebra finch sample pairs from project GSE34819 [32] and performed ANOVA group comparisons between samples from Area X (GSM855822, GSM855826, GSM855830, GSM855838, GSM855842, GSM855846, GSM855850, and GSM855854) and the ventral striato-pallidum (VSP; GSM855823, GSM855827, GSM855831, GSM855839, GSM855843, GSM855847, GSM855851, and GSM855855). Examples of the brain regions laser captured for RA vs. VLA, and Area X vs. VSP are presented in Pfenning, [23] (Fig. S17) and Hilliard, [32] (Fig. 1), respectively.

#### Removal of non-informative, low-signal, and high-variance cDNAs/oligos

Previous efforts aligned the ESTs/cDNAs and their corresponding 60-mer oligonucleotides to the zebra finch genome (taegut1) and annotated each oligonucleotide based on its proximity to ENSEMBL gene model predictions (Pfenning et al. [23]; Whitney et al.[28]). However, these efforts did not investigate or confirm the suitability of individual cDNA/oligo probes for evaluating a single gene’s expression. Based on our experience with these microarrays, there are numerous cases where cDNAs from the corresponding clone collections result from cloning artifacts (e.g. second strand oligo-dT priming), and other cases where the EST or oligo sequences align to multiple loci in the zebra finch genome, and thus would not be expected to reliably discriminate expression between such loci. Therefore, to minimize the impact of such non-informative cDNAs or oligos, we performed extensive curatorial efforts of the ESTs and oligo sequences associated with the ESTIMA:SoNG microarrays, and of the Agilent 45 k and 25 k oligo arrays. We removed every cDNA or oligo associated with an EST that contained a stretch of 15–20 “T’s” at the 5′-end, since these clones nearly always represent a second strand synthesis priming artifact. We next aligned the remaining EST and oligo (60-mer) sequences to the zebra finch genome (Taegut1) using BLAT [42] with stringent alignment parameters (minScore = 30; minIdentity = 0). As a first pass, we removed every EST or oligo that failed to align to the finch genome (i.e. minimum identity score of 95% for ESTs, and alignment score of 25 for oligos), since it is not currently possible to assess the specificity of these probes or to establish an accurate transcript orthology based on synteny analysis. We next removed any ESTs or oligos that aligned to two (or more) loci that were on different named (or known) chromosomes. ESTs and oligos that had secondary alignments to Chr_Un with high sequence identity scores (> 95%) were retained since these secondary loci are presumed to correspond to allelic loci. This screening effort resulted in the removal of a large number of unsuitable oligos (~ 5500, i.e. 13% of the non-redundant oligos) and cDNAs (~ 1500, i.e. 7% of ESTs), as summarized in Additional File 1: Table S1.

For each experiment conducted in the oligo array platform, we removed sets of oligos that gave very low normalized intensity values, since these oligos essentially failed to detect expression, and thus are non-informative. Specifically, for each experiment we ranked the entire set of oligos from high-to-low according to the normalized average expression value measured for each sample type in each pair of samples (*n* = 3 samples per region). We then plotted the average signal intensity versus an oligo’s ranking and examined the resulting curve to determine whether there was an obvious inflection or shoulder in the distribution indicating a lack of detectability in the signal (Additional File 2: Fig. S1A-C). This inflection point was used to establish minimum signal detection cut-off limits for each experiment. Specifically, we removed oligos that had an average maximum intensity value less than the average value at the inflection point plus 2.5 times the standard deviation of that value. We next identified oligos that had very high signal variance and thus would not contribute reliable information about the regulation of genes represented by multiple oligos on the array. Oligos with a stdev/mean value two times greater than the average stdev/mean for the entire population of oligos were removed from further analysis. On average, these steps combined resulted in the removal of ~ 3-10 k oligos per experiment (summarized in Additional file 1: Table S1).

### Establishment of Significance Cut-offs based on ZEBrA expression data

**Fig. 2:**
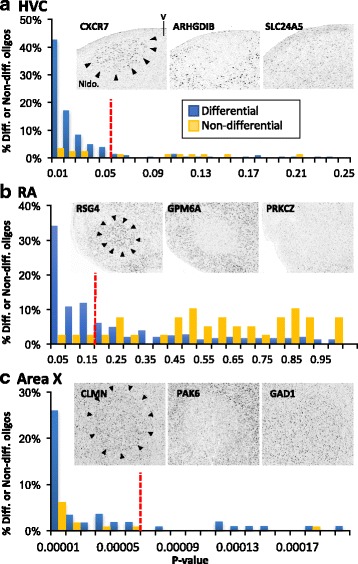
Establishment of Significance Cut-offs For HVC, RA, and Area X Array Studies Based on an Analysis of Gene Expression Patterns in the Zebra Finch Brain Expression Atlas (ZEBrA). Differential (left and middle photomicrographs in A-C) and non-differential genes (example photomicrographs in A-C) were identified for each song nucleus (A-C) by visually analyzing a subset of 320 high resolution in situ hybridization images from the ~ 500 that are available on the ZEBrA finch expression brain atlas (ZEBrA; www.zebrafinchatlas.org; in situ gene lists in Additional File 1: Table S2). For each nucleus, *p*-value frequency histograms were calculated for sets of oligo that corresponded to differential or non-differential genes, and the histograms were plotted on the same graphs (graphs in A-C). High-confidence *p*-value cut-offs (dotted lines in the graphs in A-C) were established visually and define p-value cutoffs that capture the highest proportion of true vs. false positives. *P*-value cutoffs were used to define significant versus non-significant oligos in the subsequent oligo array scoring analysis (see methods for details)

Efforts to identify significantly differential genes based on microarrays typically rely upon ‘blind’ analyses using ANOVA statistics with false-discovery rate correction. While informative, we have been able to improve on this approach by analyzing high resolution *in situ* hybridization images available on the Zebra finch Expression Brain Atlas (ZEBrA; www.zebrafinchatlas.org) for differential and non-differential genes, to establish validated *p*-value cut-offs for each anatomical comparison. We first examined expression for the entire set of genes with *in situ* hybridization data available at ZEBrA (n = ~ 490 genes) and identified 320 genes where the data allowed us to assess each nucleus of interest and its adjacent tissue. For each nucleus (with the exception of nXIIts) we then defined subsets of genes that were either differential (high or low) or expressed but non-differential, when compared to the respective adjacent regions of contrast (photomicrographs in Fig. 2a-c). A summary of the subsets of differential vs. non-differential genes that were analyzed for each nucleus is presented in Additional file 1: Table S2. In cases where no signal was detected in either brain region, labeling was inconsistent within or between sections, folds or artifacts were present that obscured evaluation, or the presence of the nucleus or its anatomical comparison could not be established, the *in situ* data for that gene was removed from further analysis. For each nucleus, we next retrieved the *p*-values for every oligo on the array that corresponded to a gene from the differential and non-differential gene sets. We then constructed p-value frequency histograms (Fig. 2a-c) for each of these gene sets, and plotted both differentials and non-differentials on the same graphs. We then established a high-confidence cut-off corresponding to a p-value that captured the highest proportion of true vs. false positives (vertical dotted lines in graphs in Fig. 2a-c). These p-values were used as validated cut-offs to define the universe of oligos that were analyzed in the subsequent scoring analysis.

#### Oligo microarray curation and scoring

After sorting the set of oligos/cDNAs from each respective microarray analysis according to their p-value, we next applied the validated *in situ*-based p-value criteria and retrieved initial core sets of significant oligos. Before proceeding with the analysis, however, it was first necessary to fully annotate these sets of significant oligos through an extensive curation effort. As briefly discussed above, Pfenning et al. [23] and Whitney et al. [28] provided an initial set of annotations for the Agilent oligonucleotides by aligning ESTs and their corresponding 60-mer oligonucleotide sequences to the zebra finch genome (Taegut1). They next annotated each oligo according to its relative proximity to ENSEMBL gene model predictions. More specifically, ESTs that mapped to within 3 kb (or oligos within 5 kb) of an ENSEMBL model were assigned the annotation given to that ENSEMBL model. However, based on a careful analysis of these annotations, we discovered numerous cases where this approach did not to take into account strand information, did not detect ESTS/oligos that were clearly intronic to a gene model, failed to correctly assign an EST to an overlapping gene model, or assigned ESTs/oligos to an ENESMBL model that was itself incorrectly annotated. In addition, we have identified numerous cases where an EST was derived from a cDNA that is clearly a product of reverse transcription priming or cloning artifacts so that the resulting oligo assesses the wrong strand with reference to the gene. To address these issues, we applied a curatorial pipeline that has previously been described in [25, 43, 44] in order to annotate and in some cases, correct the annotations of oligos/ESTs. The approach consists of BLAT aligning oligo and EST sequences against the zebra finch genome and then annotating these sequences based on their association with a gene model on the correct strand (i.e. zebra finch Ensembl prediction or Refseq, or in some cases Xenorefseq). In many cases, we also verified gene orthology by performing additional BLAT/BLAST alignments and synteny comparisons with other avian (i.e., chicken) and non-avian genomes (i.e., alligator, lizard, mouse, human). This approach was applied as needed in order to annotate each significant oligos/ESTs that was unannotated, and also to address specific cases such as those described the paragraph below.

Next, we retrieved from each experiment the full set of ESTs/oligos that had the same gene annotations as the initial core sets of significant ESTs/oligos. Many of these additional ESTs/oligos presented the same issues as discussed above, which could be easily verified since many of these additional ESTs/oligos had the same gene annotations but had different chromosomal locations or different strands. Thus, an additional round of curations was applied, essentially as described in the preceding paragraph. Through this iterative curation process, we were able to generate highly-reliable, complete sets of significantly differential oligos for each region analyzed. The re-annotated oligo sets that helped us to derive the present conclusions about differential and non-differential genes that are presented in Additional file 1: Tables S3–6 for each song nucleus analyzed. We note that our efforts in this and other studies resulted in further evaluation and/or reannotations of a total of > 9300 oligos the Agilent arrays (see Additional file 1: Table S1). The full set of these reannotations, beyond those presented in Additional file: Tables S3-S6, does not impact the present results and will be presented in a separate report.

Our significance cut-off analysis revealed many cases where multiple oligos probing the same gene had inconsistent *p*-values, as well as cases where multiple oligos were probing an identical region of a gene (> 75% overlap at the nucleotide level), and thus these oligos cannot be considered as independent measures of gene expression (example in Additional file 2: Fig. S2). To address these issues, we developed a majority rule scoring assessment based on counts of unique significant and non-significant oligos associated with each gene. Specifically, for each gene, we examined the complete set of oligos associated with that gene, and counted separately the number of oligos that had p-values above and below the p-value cut-off as established by the analysis of ZEBrA expression data as described above. In cases where multiple oligos were found to be probing the same nucleotide sequence, we averaged *p*-values, and only counted that sequence once in the scoring. In cases where > = 50% of oligos were below the *p*-value cutoff the gene was scored as differential; in all other cases the gene was scored as non-differential.

### Bioinformatics

#### Pathway over-representation analysis for individual nuclei

To identify possible molecular and biochemical pathways, as well as protein and gene interactions among differential marker sets we conducted an over-representation analysis using ConsensusPathDB (CPDB; http://cpdb.molgen.mpg.de/; [45]). For each nucleus, we analyzed the differential gene set against a background set consisting of the complete set or “universe” of genes assessed in that experiment. Over-representation analysis was conducted against the full collection of pathways available in CPDB using standard parameters (minimum overlap = 2; *p*-value cutoff = 0.01). The complete lists of enriched pathways for each nucleus are presented in Additional file 1: Tables S7–10. For the data presented in Tables 2-4 we further curated each set of pathways with a corrected q-value < 0.05, and eliminated redundant pathways and/or pathways defined only by generic terms. An over-representation analysis was also conducted against the complete set of level 5 Gene ontology terms and these results are presented in Additional file 1: Tables S11–14.

#### Shared marker and pathway over-enrichment analyses for the song system

Because the universes of analyzed genes differed across experiments, we first identified the subset of genes that were assessed in all 4 experiments to define a common universe of assessed genes. Next, we used a Venn diagram [46] to define the sets of markers that were shared among all nuclei, shared among nuclei of the DMP, and shared among the forebrain song nuclei. We note that this analysis removed any differential markers of specific nuclei that were not present in the common universe of assessed genes. To gain further insights into shared pathways, we then performed a pathway over-representation analysis on the overlapping sets of markers using an approach identical to that applied to the individual nuclei as described above.

#### Construction of gene family figures

To construct the gene family figures (Figs. 4, 5, 6, 7, 8 and 9), we first identified sets of genes involved in a variety of functions of potential interest to understanding the physiology and modulation of the song control circuitry. These sets of genes were identified based on a combination of literature searches, and gene family membership derived from the Human Genome Nomenclature Consortium database. For each gene, we performed orthology verification (as described above), and determined whether each gene was differential by microarray (shaded in green or red to indicate + or – regulation), non-differential (shaded in yellow), or not assessed (shaded in grey) for each song nucleus examined, as indicated in Figs. 4, 5, 6, 7, 8 and 9. Importantly, we noticed that in spite of our extensive curatorial efforts, several genes on Figs. 4, 5, 6, 7, 8 and 9 were still associated with mis- or unannotated oligos, leading to errors in determining the occurrence and/or direction of their differential expression. This issue was particularly relevant for oligos that were not considered significantly differential in any of the 4 experiments in this study, and whose annotations had therefore not been verified. To address this issue, we constructed a BED-track consisting of the genomic position and direction of differential regulation (or lack thereof) of each oligo that assessed in each of the 4 differential screening experiments. Uploading these BED-tracks to UCSC’s zebra finch genome browser allowed us to systematically verify the annotation of each oligo associated with a locus and in some cases, correct the microarray scoring for the genes presented in Figs. 4, 5, 6, 7, 8 and 9 (see examples in Additional file 2: Figure S2). This was conducted in parallel with synteny verification for orthology determination), and led to the further curation of hundreds of additional mis- or unannotated oligos that would otherwise have escaped our attention.

To complement our assessments of differential gene expression by microarrays we next analyzed *in situ* hybridization data for an additional 60 genes that were not part of set analyzed to establish *p*-value cutoffs (Figs. 4, 5, 6, 7, 8 and 9). This included 6 genes that were not assessed in any nucleus by microarrays. This effort allowed us to determine the occurrence and direction of differential regulation for genes that were not assessed by the microarrays or whose corresponding EST/oligos had been removed for technical reasons during the curatorial stages of the microarray analyses. In cases of conflict between the microarray and *in situ* data, we report the outcome of the *in situ* analysis since it provides a more rigorous and validated approach for verifying differential expression. Members of the gene families that were not assessed by microarray or by *in situ* hybridization are not presented in Figs. 4, 5, 6, 7, 8 and 9. We note that song nuclei DLM, LMAN, and DM were only assessed by *in situ*, and that only a few genes were assessed by *in situ* for nXIIts.

#### In situ hybridization

A total of 380 genes were analyzed by *in situ* hybridization (ISH) to establish high-confidence p-value cut-offs for the analysis of microarray data (*N* = 320 genes) and expand the data set for assessing the song system transcriptome (*N* = 60 genes). We note that the *in situ* hybridization data are derived from a minimum of 2–3 hybridizations for each gene, and high-resolution digital images for most of these genes are available in the Zebra finch Expression Brain Atlas (www.zebrafinchatlas.org). Unless otherwise indicated, all probes used are derived from the Songbird Neurogenomics consortium zebra finch brain cDNA collection [37]. A complete description of the protocol for conducting *in situ* hybridizations on zebra finch brain sections, including animal preparation, brain histology, preparation of bacterial clones and cDNA templates, synthesis of DIG-labeled riboprobes, hybridization and post-hybridization washes, immunohistological detection of DIG probes, hybridization controls (e.g. probe and antibody omission, sense and antisense probes), chromagen development, and section imaging and processing can be found in [47] (see also [25, 48]). We used GIMP or Photoshop CS5 (Adobe; San Jose, CA) to adjust the contrast, brightness, and greyscale balance of images, and also to correct for any artifacts introduced during slide processing (e.g. scratches, chromagen artifactual precipitate). Figures were prepared in Powerpoint v15 (Microsoft; Redmond, WA) or Illustrator CS5 (Adobe; San Jose, CA).

#### Probe Selection and Specificity

For every gene analyzed in this study by *in situ* hybridization we have selected a single cDNA clone that is partially overlapping and/or contiguous with the predicted Ensembl gene model based on a series of overlapping EST reads. To ensure probe specificity, these clones generally consist of non-coding or 3′-untranslated (3-UTR) EST sequence since these regions typically do not contain coding regions that are shared by other members of the same gene families. In a small number of cases, we have selected clones that are non-overlapping but found to align within ~ 500 bp of the 3′-end of a gene model. Given the close proximity of these reads to the gene model, evidence of polyadenylation (i.e. poly-A tail), and correct orientation to the +/− DNA strand, we have concluded that these cDNAs most likely correspond to the 3′-end of the gene. To further confirm clone specificity, we perform BLAT alignments of corresponding EST sequences to the zebra finch genome. The majority of ESTs align unambiguously to a single locus. In a small number of cases an EST may align to secondary loci, but the secondary alignment scores are typically much lower, indicating a high likelihood of probe specificity. Finally, in cases where no ESTIMA clones are available for a given gene, we have generated template by RT-PCR cloning.

## Results

### Microarray Analysis and Probe Curation

We conducted a differential screening analysis to identify molecular specializations of 4 major song nuclei (HVC, RA, nXIIts, and Area X) using data from cDNA and oligo microarrays hybridized with laser-capture samples from these nuclei and adjacent areas. Although we made use of datasets that are publicly available in GEO, an extensive curatorial effort was necessary to remove a large number of systematic errors present in these datasets. First, to remove unreliable probes, we aligned the sequences for the complete sets of ESTs and oligos on the microarrays to the current zebra finch genome assembly (*taegut1;* WUGSC 3.2.4; [49]). This step revealed that 5518 (~ 13%) oligos and 1507 (~ 8%) ESTs were unreliable due to: (a) multiple high-scoring alignments, indicating a lack of specificity, (b) non-informative alignments (i.e. low-scoring or no alignment, or alignments exclusive to chromosome Unknown) that precluded gene identification, or (c) evidence of artifacts of mis-priming or cloning in the construction of the cDNA databases (Additional file 1: Table S1). All such problematic oligos and ESTs were removed from subsequent analyses.

At several stages of our analysis we noticed that a large number of oligos were either unannotated or mis-annotated. We therefore performed an extensive manual curation effort and directly examined the annotations for 9305 oligos (~ 22% of oligos on the oligo array; Additional file 1: Table S1). Of these, we corrected 2735 annotations that were incorrect (~ 29% of the oligos examined), updated the names of 2440 genes to current HGNC gene terms, and identified 1466 differential oligos with unknown orthology, including several that possibly represent novel genes within the songbird lineage. A complete analysis of the unknown subset will require further curatorial efforts, and will be presented in a separate study.

Next, after ranking the remaining oligos/cDNAs according to their *p*-values for each experiment, we removed oligos that had very low signal (Additional file 2: Figure S1A-C) and/or very high variance, typically amounting to 3000–10,000 oligos per experiment (see Additional file 1: Table S1 for a breakdown by nucleus). We then carefully analyzed the large *in situ* hybridization database available on ZEBrA and plotted p-value histograms for the proportion of oligos associated with genes that had differential versus non-differential expression in HVC, RA, and Area X (blue and orange columns, respectively in Fig. 2a-c). This effort allowed us to identify the range of histogram bins containing high proportions of true positives (differential by *in situ* and microarrays) and low proportions of false positives (differential by microarray, but non-differential by *in situ*), and thus define *in situ* validated significance cut-offs (vertical red dashed lines in Fig. 2a-c). Brainstem nucleus nXIIts could not analyzed in this manner due to a lack of available in situ data, thus we used a standard *p* < 0.05 significance cut-off for that screening. Altogether, our efforts generated large sets (~ 600 to ~ 1800) of high-confidence, known protein coding genes that are up- or down-regulated in major song nuclei compared to adjacent tissues (Table 1; for full lists of genes that were differential, non-differential, or not assessed in each nucleus see Additional file 1: Tables S3-S6).

**Table 1:** Breakdown of differential and non-differential genes, and over-represented pathways in the song system

	HVC vs. Shelf	RA vs. VLA	nXIIts vs. SSP	Area X vs. VSP
Genes Assessed	7256	8679	9034	6441
Enriched (+)	341	853	380	133
Impoverished (−)	376	926	779	485
Total Markers (+/−)	717	1779	1159	618
Bioinformatics				
Over-represented Pathways	98	35	93	67
Over-represented level 5 GO terms	92	34	86	55

### Gene Over-representation Analysis

Over-representation analysis of the differential gene sets revealed distinct sets of pathways and Gene Ontology (GO) terms that are significantly overrepresented in each nucleus (summarized in Table 1; full lists of significant enrichments in Additional file 1: Tables S7–10, and GO term enrichments Additional file 1: Tables S11–14). In general, the number of over-enriched pathways and GO terms per nucleus was proportional to the number of differential genes for that nucleus. A notable exception was RA, which had nearly twice as many differential markers as HVC, but only ~ 1/6th (or fewer) enriched pathways and GO terms. Also of note, ‘neuronal function’ and ‘neuroactive receptor activation’ were the only specific pathway annotations shared by all nuclei (Table 2A and B). This was due to a large complement of ion channel, neurotransmitter, and axon guidance related genes in the former, and myriad neuropeptide, cannabinoid, opioid, and purinergic receptors in the latter that were differential markers of all song nuclei. However, closer examination revealed that the specific genes underlying these shared annotations differed markedly across nuclei. For example, numerous but distinct potassium channel genes are differential markers of the nuclei in the DMP, but not Area X. This is consistent with the more specific pathway annotation ‘voltage-gated potassium channel’ being enriched in all DMP nuclei, but not in Area X, and is supported by a previous report on the expression of potassium channel-related genes in the song system [25].

**Table 2:** Summary of major pathways over-represented in each song nucleus

HVC	RA	nXIIts	Area X
A. Ion Channels and Cellular Excitability			
Cardiac conduction	Cardiac conduction	Cardiac conduction	Cardiac conduction
Voltage gated K+ channels	Voltage gated K+ channels	Voltage gated K+ channels	Phase 0 - rapid depolarization
2 pore K+ channels	Inactivation of Na + channels		Phase 2 - plateau phase
	Resting membrane potential		
B. GPCR Signaling			
GPCR Adenosine A2A receptor	GPCR signaling-G alpha q	GPCR signaling-cholera toxin	G alpha (i) signaling events
GPCR Dopamine D1 receptor	GPCRs, Class A Rhodopsin	GPCR signaling-G alpha i	
		GPCR signaling-G alpha q	
		GPCR signaling - Epac and ERK	
		GPCR signaling - PKA and ERK	
		GPCR signaling-pertussis toxin	
		Secretin family receptors	
C. Neuromodulatory Systems			
Cholinergic synapse	Biogenic Amine Synthesis	Amphetamine addiction	Adrenergic signaling
		Cholinergic synapse	Dopamine Release Cycle
		Dopaminergic synapse	Serotonin signaling
		Serotonin and anxiety	Beta-agonist/Beta-blockers
D. Intracellular signaling			
Phospholipase D signaling	cAMP signaling pathway	Phospholipase D signaling	cGMP-PKG signaling pathway
CREB phos. Via CaMKII	cGMP effects	PLC beta mediated events	rho-GEF activation
Ras signaling pathway		PLC-gamma1 signaling	
		DAG and IP3 signaling	
		cGMP-PKG signaling	
E. Lipid Metabolism			
HDL-mediated lipid transport	Glycerolipid metabolism		Glycosphingolipid biosynthesis
Lysosphingolipid receptors	Phospholipid Biosynthesis		Lysosphingolipid receptors
triacylglycerol biosynthesis	lipoprotein lipase deficiency		
	Glycerol Kinase Deficiency		
F. Growth Factor Signaling			
NGF signalling via TRKA	FGFR1 ligand binding	Signaling by EGFR	
Signaling by EGFR	FGFR2c ligand binding	Signaling by VEGF	
Signaling by PDGF		Internalization of ErbB1	
Signalling by NGF			
VEGF Signaling Pathway			
VEGFR2 mediated prolif.			
G. Axon guidance and cell-cell interaction related			
Axon guidance		Axon guidance	Axon guidance
neurexin/neuroligin complex		neurexin/neuroligin complex	Adherens junctions interact.
ECM-receptor interaction		Reelin signalling pathway	NCAM1 interactions
Integrin			Netrin-1 signaling
LGI-ADAM interactions			Cell adhesion molecules
neurite out-growth (NCAM)			CRMPs in Sema3A signaling
H. Glutamate transmission			
Activation of NMDA receptors		Activation of NMDA receptors	Glutamatergic synapse
		Trafficking of AMPA receptors	
I. Calcium Signaling			
Calcium signaling pathway		Calcium signaling pathway	Calcium signaling pathway
CAM-kinase activation		CAM-kinase activation	Reduction of cytosolic Ca2+
		Calmodulin induced events	Sodium/Calcium exchangers
		Elevation of cytosolic Ca2+	
J. Peptide signalling			
Gastrin-CREB signaling		Gastrin-CREB signaling	Peptide binding receptors
Oxytocin signaling pathway		Oxytocin signaling pathway	NFAT signaling in lymphocytes
Renin secretion		Opioid Signalling	Morphine addiction
		Vasopressin regulation	
		Morphine addiction	
K. Specific Pathways Shared by two or more song nuclei			
Hemostasis		Hemostasis	Hemostasis
Circadian entrainment		Circadian entrainment	Circadian entrainment
Keratan sulfate biosynthesis			Keratan sulfate biosynthesis
Rhodopsin-like receptors			Rhodopsin-like receptors
Gap junction		Gap junction	
nitric oxide signaling		nitric oxide signaling pathway	
Insulin secretion		Insulin secretion	
Long-term potentiation		Long-term potentiation	
Taste transduction		Taste transduction	
		Aldosterone secretion	Aldosterone secretion
		Purine/Pyrimidine metabolism	Purine/Pyrimidine metabolism
		Olfactory transduction	Olfactory transduction
L. Pathways Unique to Individual Song Nuclei			
Amyotrophic lateral sclerosis	Cell cycle: g2/m checkpoint	Aquaporin-mediated transport	Gene reg. by peroxisome proliferators
Angiogenesis overview	Amino acid metabolism	CLEC7A/NFAT activation	
Chemokine/DAP12 signaling	somitogenesis	NDK phosphins and dynamin	
Glycosaminoglycan metabolism	Glial Cell Differentiation	FTO Obesity Mechanism	
RET signaling	peroxisomal oxidation	MNGIE disease	
Toxicity of botox A		Nicotin-ate/−amide metabolism	
		Regulation of pgc-1a	
		endocannabinoid signaling	
		Salivary secretion	
		Signaling by Wnt	

Other broad pathway annotations were shared by all nuclei except for RA, reflecting the much smaller number of specific pathways present in the latter. These included G-protein coupled receptor (GPCR) signaling, calcium and non-calcium intracellular signaling, and axon guidance (Table 2C-F). However, while these nuclei all had pathways within these general categories, no specific pathways were shared across all nuclei. For instance, GPCRs for ‘metabotropic glutamate receptors’ were exclusively featured in HVC, ‘rhodopsin-like receptors’ were shared features of HVC and Area X, and nXIIts features no fewer than 6 different G-protein related receptor pathways. Lastly, other specific pathways were uniquely over-represented in individual nuclei (Table 2K). Noteworthy are the glycosaminoglycan pathway for HVC, and a broad diversity of signaling and metabolic pathways for nXIIts.

**Fig. 3:**
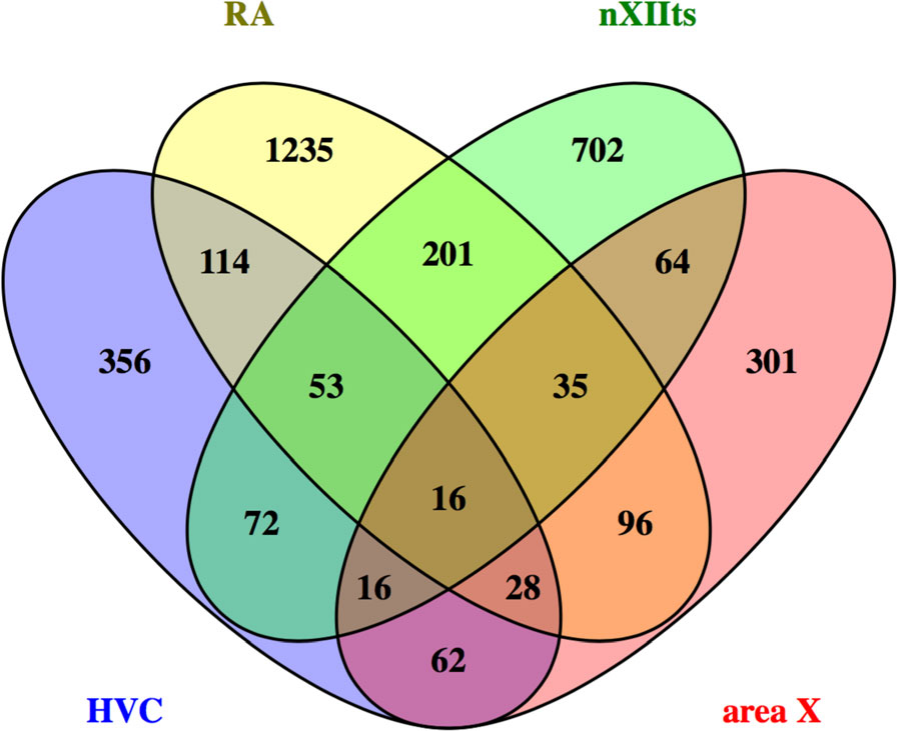
Venn Diagram of the Differential Markers Regulated in HVC, RA, nXIIts, and Area X. 16 genes were common to all song nuclei, 53 were common to the DMP, and 28 were shared markers of telencephalic nuclei

To gain further insights into features unique to individual nuclei or shared across nuclei, we constructed a Venn diagram with lists of differential genes for each song nucleus and determined all possible intersections (Fig. 3; gene sets from the main intersections are presented in Additional file 1: Table S15). This analysis revealed that collectively the nuclei shared 16 (~ 1%) markers, which were enriched for ‘cardiac conduction’ and ‘potassium channels’, as well as pathways related to molecular interactions at synapses (Table 3A). These results differed significantly from analysis of the marker set shared by telencephalic nuclei, which showed enrichments in signaling and structural pathways (Table 3B), as well as from analysis of markers shared by all DMP nuclei, which showed enrichments in reelin-related pathways and calcium- and IP3-related signaling pathways (Table 3C). In contrast, the majority (70%) of markers were unique to individual nuclei (Fig. 3), reflecting a high degree of molecular specialization across nuclei. Close examination of related pathways (Table 4) showed enrichments related to sugar and amino acid metabolism in HVC (Table 4A), inwardly-rectifying potassium channels but otherwise a paucity of enriched pathways in RA (Table 4B), and a diversity of signaling and structural pathways in nXIIts (Table 4C) and Area X (Table 4D).

**Table 3:** Summary of major pathways over-represented in the song system

A. Pathways shared by all nuclei
Cardiac conduction
Muscle contraction
Potassium Channels
Parkin-Ubiquitin Proteasomal System pathway
Class A/1 (Rhodopsin-like receptors)
Interactions of neurexins and neuroligins at synapses
B. Pathways shared by telencephalic nuclei
CDC42 signaling events
Regulation of Actin Cytoskeleton
PDGFR-beta signaling pathway
RHO GTPases Activate WASPs and WAVEs
RAC1 signaling pathway
C. Pathways shared by nuclei of the DMP
Reelin signalling pathway
Signaling by GPCR
VLDL interactions
Calmodulin induced events
CaM pathway
Stathmin and breast cancer resistance to antimicrotubule agents
Ca-dependent events
Endochondral Ossification
ECM-receptor interaction - *Homo sapiens* (human)
DAG and IP3 signaling
EGFR interacts with phospholipase C-gamma
PLC-gamma1 signalling
Hemostasis
Lissencephaly gene (LIS1) in neuronal migration and development

**Table 4:** Over-represented pathways unique to individual song nuclei

A. Pathways unique to HVC
Glycine degradation
Toxicity of botulinum toxin type A and E
Pentose Phosphate Pathway
Glucose-6-phosphate dehydrogenase deficiency
Ribose-5-phosphate isomerase deficiency
Transaldolase deficiency
Amino Acid metabolism
Ammonia Recycling
B. Pathways unique to RA
Inwardly rectifying K+ channels
Oxidative Stress
C. Pathways Unique to nXIIts
Fatty acid elongation
Endocytotic role of ndk phosphins and dynamin
Signaling by NOTCH2
agrin in postsynaptic differentiation
MET activates PTK2 signaling
GPCR signaling-G alpha s PKA and ERK
Internalization of ErbB1
Signaling by GPCR
D. Pathways Unique to Area X
role of mef2d in t-cell apoptosis
Response to elevated platelet cytosolic Ca2+
CDO in myogenesis
mechanism of gene regulation by peroxisome proliferators via ppara
Role of Calcineurin-dependent NFAT signaling in lymphocytes
Platelet degranulation
Netrin-1 signaling
Cell adhesion molecules (CAMs)

### Gene family analysis

**Fig. 4:**
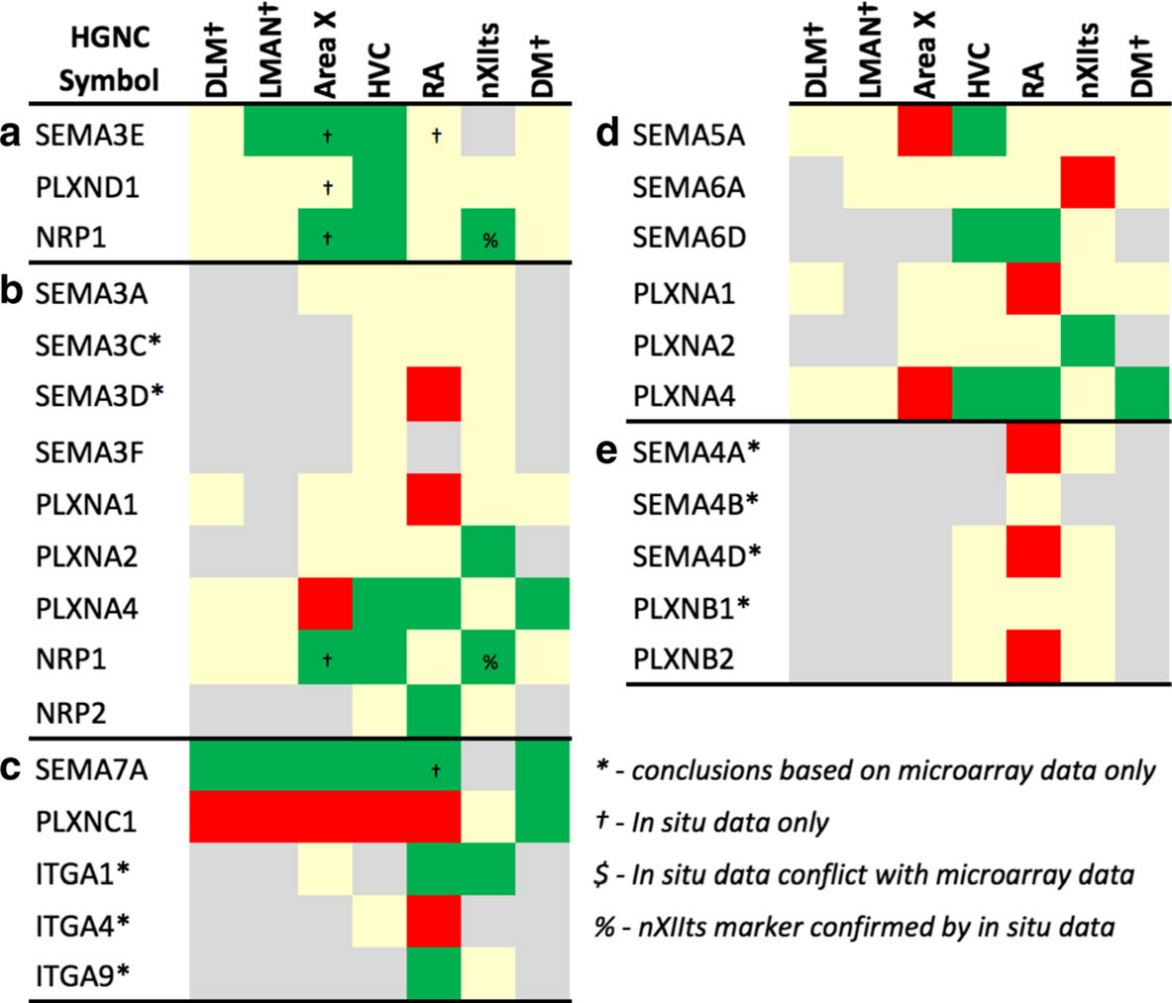
Regulation of Axon Guidance and Connectivity Genes: Plexins, Semaphorins, Neuropilins, and Integrins. Gene regulation was assessed by an analysis of microarray and/or *in situ* hybridization data available at www.zebrafinchatlas.org. Color shading indicates a gene that is differential (shaded green or red to indicate + or – regulation), non-differential (shaded tan), or not assessed (shaded grey) for each song nucleus examined. DLM, LMAN, and DM were assessed exclusively by evaluating *in situ* data

**Fig. 5:**
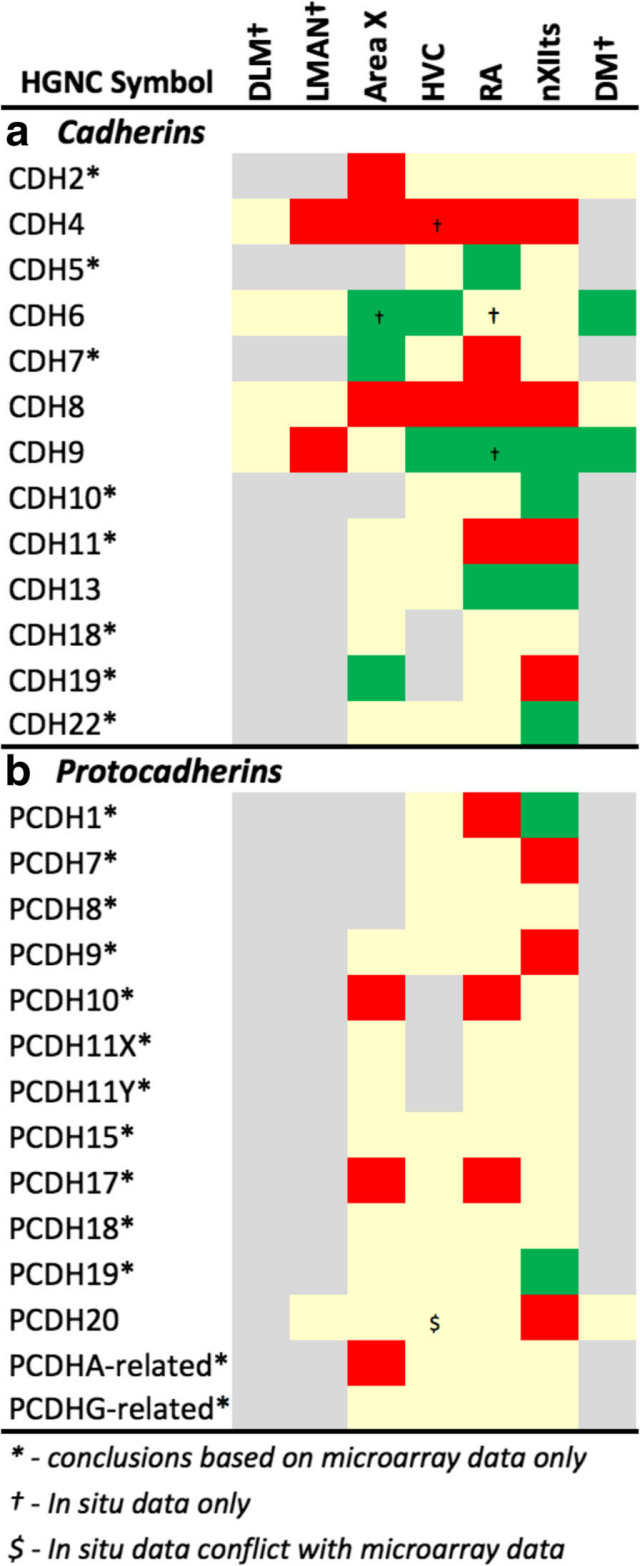
Regulation of Axon Guidance and Connectivity Genes: Cadherins and Protocadherins. Gene regulation was assessed by an analysis of microarray and/or *in situ* hybridization data available at www.zebrafinchatlas.org. Color shading indicates a gene that are differential (shaded green or red to indicate + or – regulation), non-differential (shaded tan), or not assessed (shaded grey) for each song nucleus examined. DLM, LMAN, and DM were assessed exclusively by evaluating *in situ* data

**Fig. 6:**
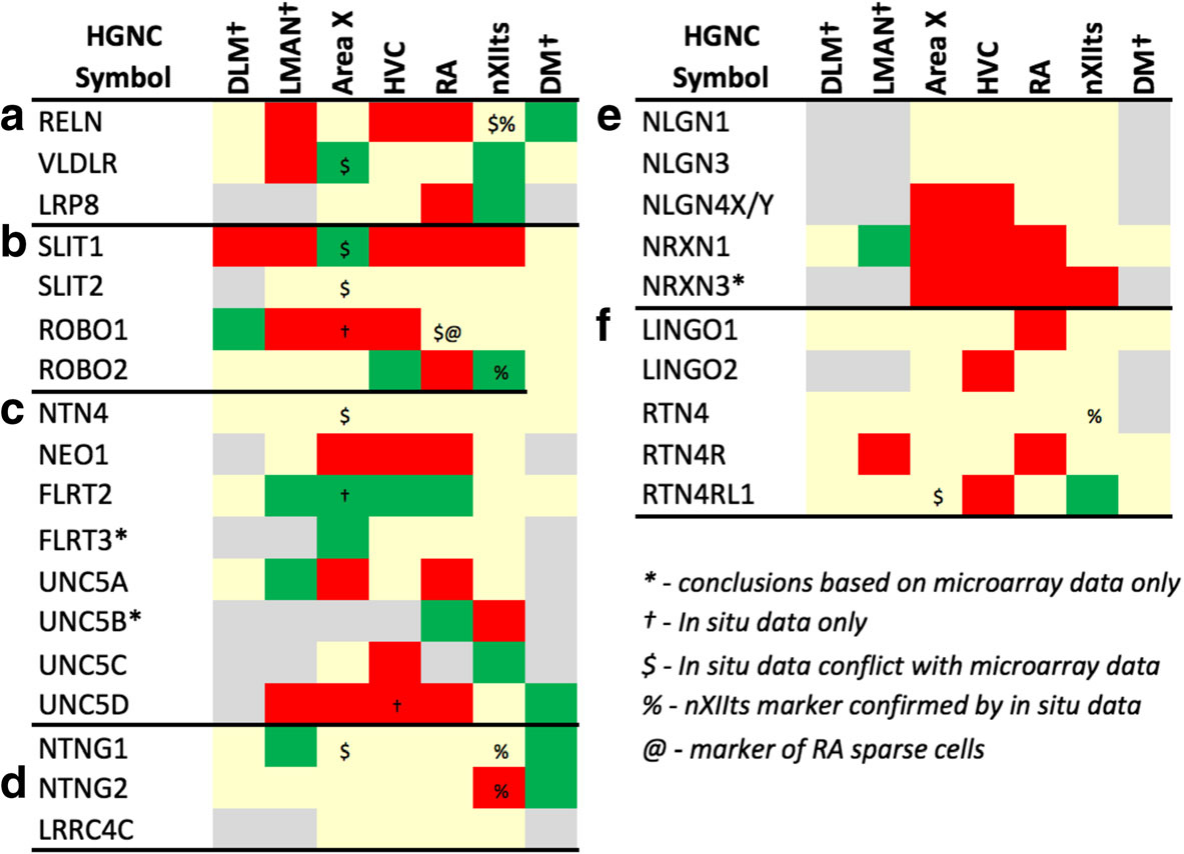
Regulation of Axon Guidance and Connectivity Genes: Netrins, Slits, and Robos. Gene regulation was assessed by an analysis of microarray and/or *in situ* hybridization data available at www.zebrafinchatlas.org. Color shading indicates a gene that are differential (shaded green or red to indicate + or – regulation), non-differential (shaded tan), or not assessed (shaded grey) for each song nucleus examined. DLM, LMAN, and DM were assessed exclusively by evaluating *in situ* data

**Fig. 7:**
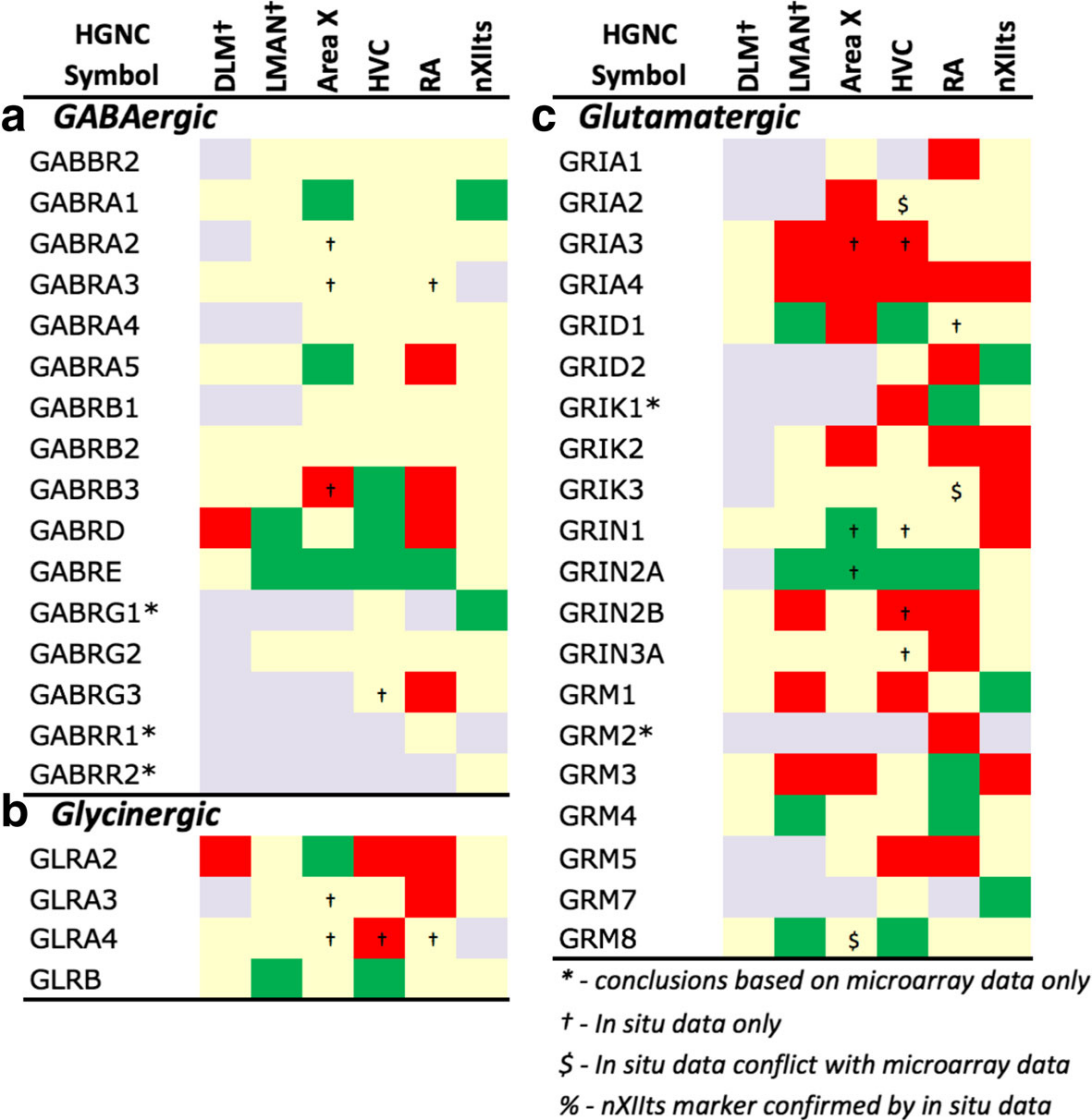
Regulation of Inhibitory and Excitatory Neurotransmission. Gene regulation was assessed by an analysis of microarray and/or *in situ* hybridization data available at www.zebrafinchatlas.org. Color shading indicates a gene that are differential (shaded green or red to indicate + or – regulation), non-differential (shaded tan), or not assessed (shaded grey) for each song nucleus examined. DLM, LMAN, and DM were assessed exclusively by evaluating *in situ* data

**Fig. 8:**
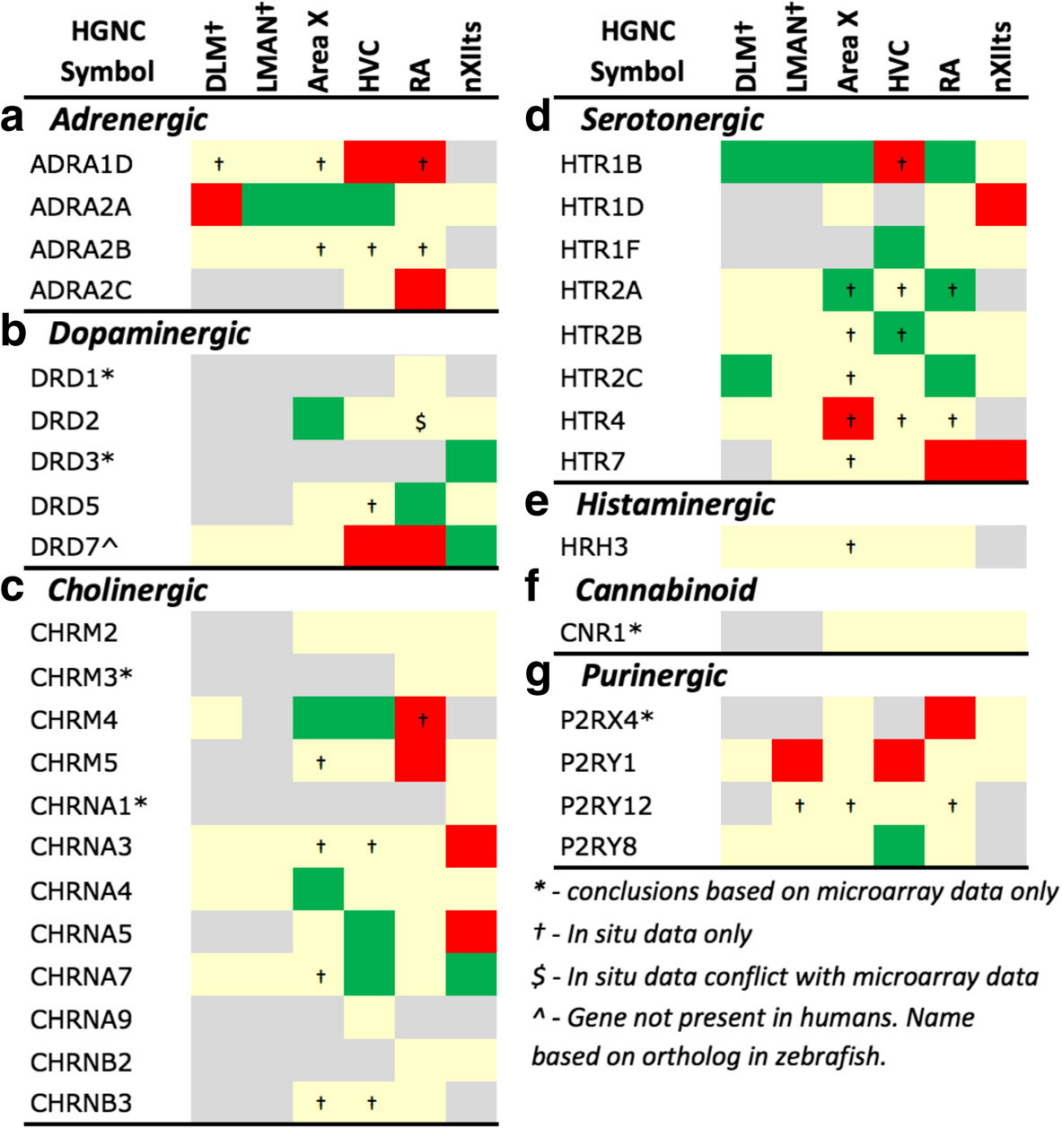
Regulation of Neuromodulatory Systems. Gene regulation was assessed by an analysis of microarray and/or *in situ* hybridization data available at www.zebrafinchatlas.org. Color shading indicates a gene that are differential (shaded green or red to indicate + or – regulation), non-differential (shaded tan), or not assessed (shaded grey) for each song nucleus examined. DLM, LMAN, and DM were assessed exclusively by evaluating *in situ* data

**Fig. 9:**
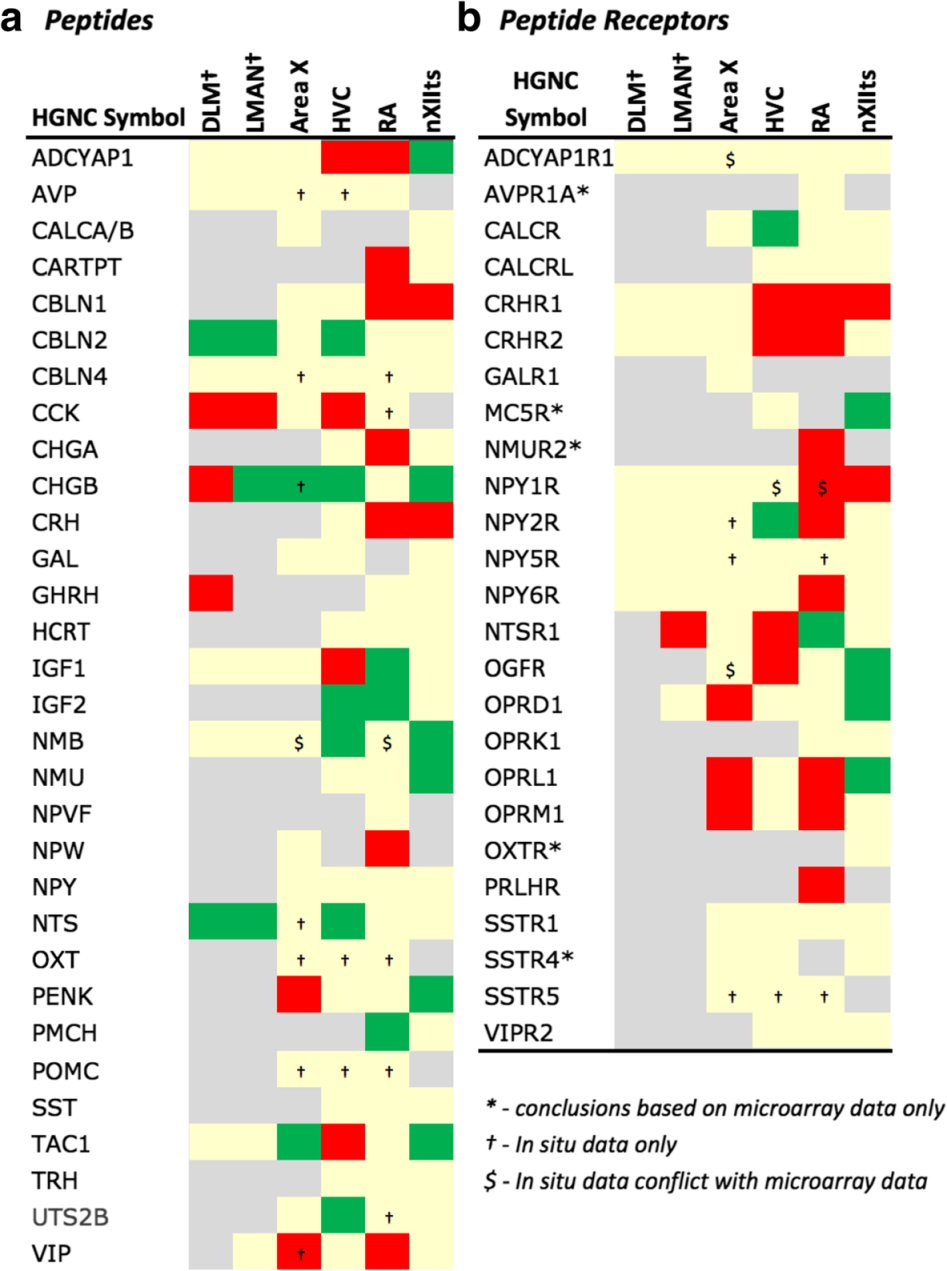
Regulation of Peptide Systems. Gene regulation was assessed by an analysis of microarray and/or *in situ* hybridization data available at www.zebrafinchatlas.org. Color shading indicates a gene that are differential (shaded green or red to indicate + or – regulation), non-differential (shaded tan), or not assessed (shaded grey) for each song nucleus examined. DLM, LMAN, and DM were assessed exclusively by evaluating *in situ* data

**Fig. 10:**
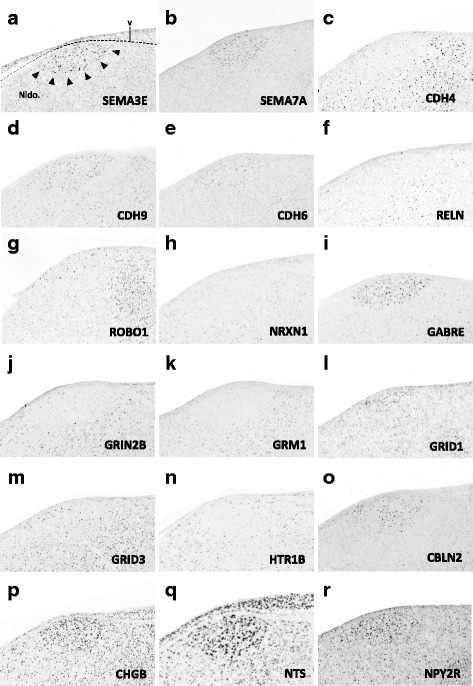
(**a–r**) Representative *in situ* hybridization photomicrographs of parasagittal sections of adult male zebra finches at the level of HVC (∼2.0 to 2.4 mm from the midline). Arrowheads in (A) approximate the ventral border of HVC. Abbreviations: nido., nidopallium; v, ventricle

**Fig. 11:**
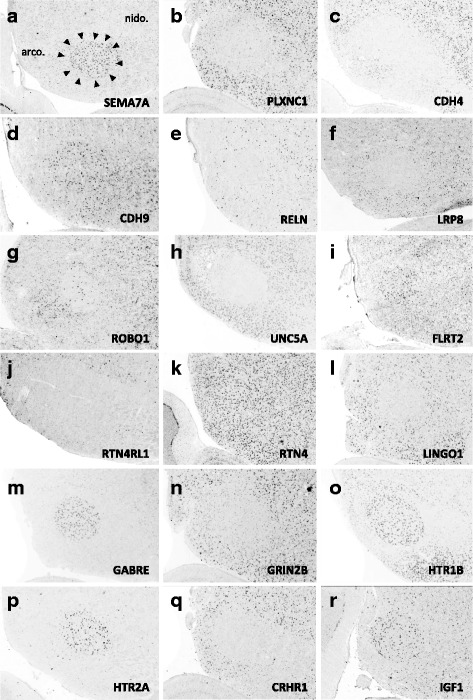
(**a–r**) Representative *in situ* hybridization photomicrographs of parasagittal sections of adult male zebra finches at the level of RA (∼2.2 to 2.4 mm from the midline). Arrowheads approximate the cytoarchitectonic borders of nucleus RA; dotted line roughly approximates the lamina between the arcopallium and nidopallium. Abbreviations: arco., arcopallium; nido., nidopallium

**Fig. 12:**
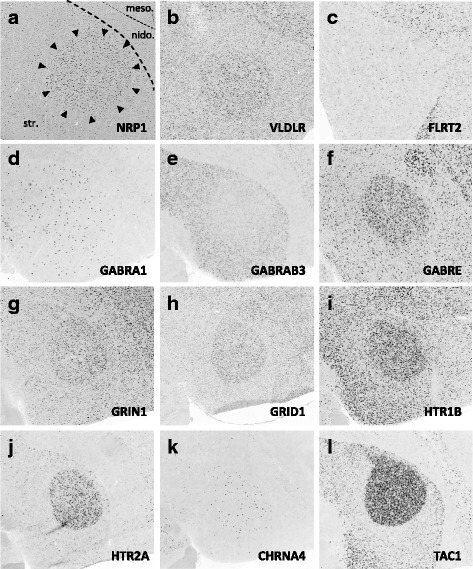
(**a–l**) Representative *in situ* hybridization photomicrographs of parasagittal sections of adult male zebra finches at the level of Area X (∼1.5 to 2.0 mm from the midline). Arrowheads approximate the cytoarchitectonic borders of Area X; dotted lines roughly approximate the lamina between the striatum, nidopallium, and mesopallium. Abbreviations: arco., arcopallium; meso., mesopallium; nido., nidopallium; str., striatum

**Fig. 13:**
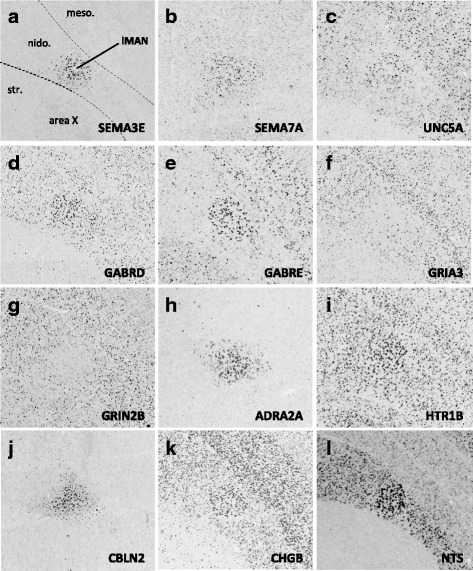
(**a–l**) Representative *in situ* hybridization photomicrographs of parasagittal sections of adult male zebra finches at the level of LMAN (∼1.7 to 2.2 mm from the midline). Dotted lines roughly approximate the lamina between the striatum, nidopallium, and mesopallium. Abbreviations: arco., arcopallium; meso., mesopallium; nido., nidopallium; str., striatum

**Fig. 14:**
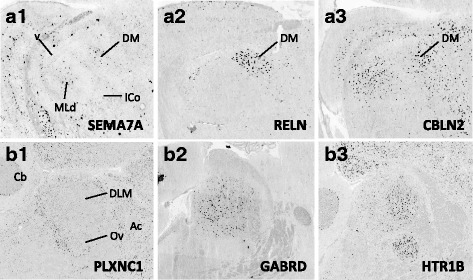
Representative *in situ* hybridization photomicrographs of parasagittal sections of adult male zebra finches at the level of DM (**a1-a3**; ∼2.4 mm from the midline) and DLM (**b1-b3**; ~ 1.0 to 1.2 mm from then midline). Abbreviations: Ac, anterior commissure; Cb; cerebellum; DM, dorsal part of the intercollicular nucleus; ICo., intercollicular nucleus; MLd, dorsal part of the lateral mesencephalic nucleus; v, ventricle

We next examined the differential expression of gene families involved in basic neuronal functions that likely influence song system organization and physiology (Figs. 4, 5, 6, 7, 8 and 9). Besides genes found by microarrays to be differentially expressed in song nuclei, we made an effort to also identify non-differential family members for a comprehensive assessment. *In situ* hybridization of available probes helped confirm the microarray data, visualize the brain-wide distribution of markers, and examine song nuclei not assessed by microarray analysis (representative *in situ* images in Figs. 10, 11, 12, 13 and 14). For some specific genes, and for LMAN, DLM, and DM, *in situs* were the only source of data. The majority of genes examined were differentially expressed in at least one nucleus, and all nuclei showed differential expression of several genes in each family. *In situs* and microarrays were largely consistent, indicating the data were robust across experiments and methods, but some divergences were also noted and are indicated in Figs. 4, 5, 6, 7, 8 and 9 by a ‘$’ symbol.

#### Axon guidance and connectivity

Gene families associated with the formation of neuronal projections, including regulators of axonal fasciculation and guidance, cell-cell recognition and adhesion, and the establishment and maintenance of synaptic connections, had multiple genes that were differentially regulated across nuclei in a mosaic-like distribution (Fig. 4, 5, and 6). RA had the largest number of differential axon guidance genes, and the majority of these genes were down-regulated in both RA (25/37) and in Area X (17/28), whereas HVC and nXIIts had similar proportions of up- and down-regulated genes within this family.

Based on the current literature, some expression patterns suggest possible ligand-receptor interactions across nuclei. Among semaphorin and plexin genes (Fig. 4), there was high SEMA3E expression in both HVC (Fig. 10a) and LMAN (Fig. 13a), while their common target Area X expressed high levels of NRP1 (Fig. 12a), the co-receptor for the SEMA3E receptor PLXND1 (Fig. 4a). HVC also highly expressed both PLXND1 and NRP1 (Fig. 4a). HVC and RA strongly expressed both PLXNA4s and NRPs, but none of the SEMAs that interact with PLXNA4 receptors were markers of telencephalic nuclei (Fig. 4b). All telencephalic nuclei were highly enriched for SEMA7A (Figs. 10b, 11a, 13b, 14a1), but negative for its receptor PLXNC1 (e.g. Figs. 11b and 14B1), whereas the alternative receptors ITGA1/9 were highly expressed in RA and nXIIts (Fig. 4c). SEMA5A and 6D were highly expressed in HVC and RA, and their PLXNA-type receptors were markers of RA and nXIIts (Fig. 4d). SEMA6A had overall low expression in the telencephalon, but was prominent in brain stem nuclei, though notably down in nXIIts (not shown). In contrast, all SEMA4s and their PLXNBs receptors were not differential or negative markers of RA (Fig. 4e).

Many cadherins/protocadherins showed differential expression in Area X, RA, and nXIIts, but few in HVC (Fig. 5). Most notably, CDH4 and CDH8 were down-regulated (Figs. 10c and 11c) and CDH9 up-regulated in most nuclei (e.g. Figs. 10d and 11d), CDH6 up-regulated in HVC (Fig. 10e) and in its target Area X, and CDHs 11 and 13 respectively down- and up-regulated in RA and nXIIts. Several other cadherins were down-regulated in RA, Area X, and/or nXIIts, but no obvious patterns seen for input-target pairs.

As for other axonal guidance cues (Fig. 6), RELN was down-regulated in HVC (Fig. 10f), RA (Fig. 11e), and LMAN (not shown), but a strongly up in DM (Fig. 14a2), whereas its receptors VLDLR and LRP8 were positive markers of nXIIts and Area X (Fig. 12b; Fig. 6a). Of note, *in situ* data (not shown) indicate that RELN is not a marker of nXIIts, but expressed in a cell group lateral to nXIIts, possibly nucleus retroambigualis. Thus, the apparent up-regulation of RELN in nXIIts based on microarrays may be due to imprecision in the brain dissection. VLDLR is also a marker of LMAN and Area X, the latter presenting a conflict with the microarray data that is likely explained by the high expression of VLDLR in nucleus accumbens, which was presumably in the VSP sample that provided the contrast for Area X. Notably, RELN’s other receptor, LRP8, was down in RA (Fig. 11d). ROBO1 was down-regulated in AFP nuclei (Area X and LMAN) as well as in HVC and RA, but highly expressed in a sparse subset of cells (Figs. 10g and 11e). Among ROBO1 ligands, SLIT1 (not SLIT2) was down in LMAN and up in Area X (Fig. 6b). ROBO2 was markedly up-regulated in HVC and nXIIts. UNC5 receptors were positive or negative markers of several nuclei (e.g. UNC5A in Figs. 11f and 13c), whereas their ligands FLRT2/3 were only positive markers of some nuclei (Fig. 6c; e.g. Figs. 11i and 12c). While little information is available on the main netrin (NTN) ligands of UNC5s, NTN4 was not differential in any nuclei. Interestingly, lipid-anchored netrin ligands (NTNGs) were differential in a few nuclei, but their LRRC4C receptor (NGL1 [50]) was not (Fig. 6d). Conversely, most NLGN ligand genes were not differentially expressed, whereas their presumed NRXN receptors were down-regulated in several nuclei (Fig. 6e; e.g. Fig. 10h). The No-Go-related receptors RTN4R and RTN4RL1 had low to no expression in most song nuclei (e.g. RTN4RL1 in RA; Fig. 11j), with up-regulation only in nXIIts. Although their presumed RTN4 ligand was not differential (Fig. 11k), the potential competitor LINGO ligands were down-regulated in HVC (not shown) and RA (Fig. 11l).

#### Inhibitory and excitatory neurotransmitter receptors

With regards to genes encoding neurotransmitter receptors (Fig. 7), inhibitory GABAergic receptor type-A subunits were broadly expressed in all song nuclei (Fig. 7a), alpha subunits being mostly non-differential, with few exceptions (e.g. GABRA1 and GABRA5 were up in Area X, the former in a sparse cell population; Fig. 12d). Other subunits, most notably beta-3, delta, and epsilon, were differentially expressed in several nuclei, albeit with different directions of regulation (examples in Figs. 10i, 11m, 12e-f, 13d-e, and 14b2). Glycine receptor subunits had detectable expression in all areas examined, including forebrain nuclei (Fig. 7b). Some alpha-subunits showed down-regulation in different nuclei and up-regulation in Area X, whereas the beta-subunit was up in both HVC and LMAN. Differential regulation was much more prevalent among glutamatergic receptor subunits, including both ionotropic and metabotropic sub-types (Fig. 7c). With the exception of thalamic DLM, all song nuclei examined showed differential regulation of numerous subunits of both sub-types. Noteworthy were the up-regulation of NMDA subunits GRIN2A in LMAN, HVC, RA, and Area X, and GRIN1 in Area X only (Fig. 12g), and down regulation of GRIN2B in HVC, RA, and LMAN (Figs. 10j, 11n, and 13g). Both AMPA and kainate subunits were generally down-regulated (e.g. GRIA3 in LMAN, Fig. 13f), but GRIK1 was up-regulated in RA. Interestingly, GRID family members had sparse labeling, including GRID1 and GRID3 in HVC (Fig. 10l and m) and GRID1 in Area X (Fig. 12h), suggestive of cell type-specific expression. Lastly, subsets of metabotropic glutamate receptors showed similar regulation, including GRM1/2/3/5 (e.g. GRM1 in HVC; Fig. 10k), all generally down, and GRM2/4/7/8, largely up or non-differential. For other sub-types, the direction of regulation varied across subunits, nuclei and pathways, or the data were insufficient to reveal a pattern.

#### Neuromodulatory receptors

Numerous subunits of catecholaminergic, dopaminergic and serotonergic receptors (Fig. 8) were differentially regulated in different nuclei of both the AFP and DMP. While the majority of adrenergic receptors were non-differential or down-regulated (Fig. 8a), ADRA2A was highly up-regulated in HVC, LMAN (e.g. Fig. 13h), and Area X. We note the general up-regulation of serotonergic receptors in pallial nuclei, most notably the up-regulation of HTR1B in most nuclei (Fig. 10n, 11o, 12i, 13i, and 14b3), but down-regulation in HVC (Fig. 10j), the marked up-regulation of HTR2A in RA (Fig. 11p) and Area X (Fig. 12j), and the down-regulation of different serotonergic subunits in nXIIts. While we acknowledge a scarcity of data on dopaminergic receptors, we note the up-regulation of DRD2 in Area X, the down-regulation of DRD7 in HVC and RA, and the up-regulation of different subunits in nXIIts. Also noteworthy is the predominant up-regulation of cholinergic receptor subunits in pallial nuclei, with CHRN5 and CHRN7 up in HVC and CHRM4 and CHRN4 up in Area X (e.g. Fig. 12k), whereas nXIIts markers were mostly down-regulated. While there was some evidence of differential expression for other classes of receptor subunits (Fig. 8e-g), markers in the purinergic receptor family tended to be down-regulated.

#### Peptides and receptors

A broad diversity of up- and down-regulated markers across different song nuclei was also seen for various peptides (Fig. 9a) and some of their respective receptors (Fig. 9b). Among noteworthy patterns, some peptides (e.g. CBLN2, CHGB, NTS) were markedly up-regulated in nuclei of the AFP (HVC in Fig. 10o-q; LMAN in Fig. 13j-l; DM in Fig. 14a3 ), CRH and related receptors were markedly down-regulated in nuclei of the DMP (e.g. CRHR1 in RA; Fig. 11o), opioid receptors were down-regulated in telencephalic nuclei of both the AMP and DMP but up-regulated in nXIIts, tachykinin 1 (TAC1) was highly up-regulated in Area X (Fig. 12l), and insulin growth factor 1 (IGF1) was up-regulated in RA (Fig. 11r). We also note that while NPY2R was up-regulated in HVC (Fig. 10r), this and other NPY receptors were down-regulated in nuclei of the DMP.

## Discussion

We have presented here a comprehensive survey of differentially expressed transcripts in the main nuclei of the song control system of quiet, unstimulated zebra finches, based on a combination of microarray and *in situ* hybridization data. While the data largely support previous findings, they also reveal large sets of previously undescribed constitutive molecular specializations of nuclei in both the direct motor pathway (DMP) and the anterior forebrain pathway (AFP). These include complements of G-protein couple receptors, neuroactive receptors, axon guidance related molecules, and voltage-gated K+ channels that were shared among, but also unique to each nucleus, as well as varying pathways related to calcium signaling, CAM-kinase activation, and neurotransmitter binding. Among the newly discovered markers are numerous members of gene families with links to the establishment and maintenance of neuronal projections, regulation of neuronal excitability, the modulation of neurotransmission and synaptic function. Together, these findings allow us to generate novel hypotheses regarding possible molecular determinants of the unique properties of the song control system.

The publically available datasets analyzed here were previously examined separately to identify singing regulated genes [28, 33, 35] and convergent specializations of analogous vocal nuclei in birds and humans [23], but not to systematically identify constitutive molecular specializations of all major song system nuclei. We note that Whitney et al. [28] analyzed baseline gene expression within individual song nuclei compared to other song nuclei, but did not analyze the expression of these same genes “outside” of the song system in areas adjacent to each song nucleus. Thus, from that analysis it is not possible to know whether or not the observed contrasting expression patterns for a given nucleus represent specialized properties of that particular nucleus, or whether they reflect global properties of the general brain region where each song nucleus is located. We also note that these previous studies provided only partial reports of the microarray data. For HVC, the robust statistical approach used here identified 717 differential genes (compared to 279 in Lovell et al. [24]). For RA, only ~ 3% of markers identified here were previously reported [23], since that study focused on the small subset of shared markers of vocal areas in birds and humans. For Area X, previous efforts focused on singing-induced genes [32, 33], with constitutive genes treated as controls and not separately analyzed. The specific data from a microarray screening for nXIIts vs. SSP [35] have not been previously reported.

Our analysis differed further from previous efforts as we used the large *in situ* hybridization database in ZEBrA as an independent method to establish rigorous and objective cut-off criteria for microarray data from each nucleus examined. We therefore did not have to rely on arbitrary cut-offs and *post hoc* validation by qPCR or *in situs*. The curatorial effort was also critical to our analysis. Since the cDNA and oligo microarrays were constructed prior to the zebra finch genome, the specificity and adequacy of individual probes had not been previously determined. By aligning each oligo/EST sequence to the zebra finch genome we were able to identify and remove ~ 5500 oligos and ~ 1500 cDNA because they had: (a) multiple high-scoring alignments, indicating a lack of specificity, (b) non-informative alignments (i.e. low-scoring or no alignment, or alignments exclusive to chromosome Unknown) that precluded gene identification, or (c) were artifacts of mis-priming or cloning. We also removed additional oligos (3000–10,000) from each differential screening because they had no detectable signal or behaved inconsistently (high variance) across arrays. Finally, our efforts revealed that ~ 29% of the oligos we examined on these arrays, originally annotated using automated approaches, present various annotation issues that greatly limit the accuracy of the data interpretation. By addressing these issues, our curatorial effort vastly improved the overall sensitivity, reliability, and accuracy of the microarray approach to identify differential gene sets. This in turn facilitated discovering molecular features unique to individual song nuclei or shared across multiple nuclei. Overall, the data represent the most rigorously vetted and annotated set of constitutive molecular specializations of the zebra finch song system performed to date.

Importantly, we found that the microarray and *in situ* data were largely concordant, but note some inconsistencies, some of which possibly reflecting differential regulation of splice variants. We also note that while the microarray data provided broad and unbiased assessments of the differential song nuclei transcriptomes, examination of gene families helped to identify targets of regulation in the context of specific pathways and categories. These general and specific insights are discussed in further detail below.

### Molecular Specializations of the Song System

Ion channel physiology, which is critical for many aspects of neuronal function, emerged from both the bioinformatics and Venn diagram analyses as a major target of regulation in the song system (Fig. 3; Table 3). This outcome is consistent with our previous findings of widespread regulation of potassium channel genes in the song system [25], and likely reflects a need for the song system to tightly regulate features of cellular excitability and spiking fidelity. The present study also reveals that members of the sodium-, calcium-, chloride-channel gene families are important targets of regulation in the song system. A comprehensive analysis of these gene families and their brain expression is being prepared as part of a separate study (Friedrich et al., in preparation). Of note, the specific complements of differential ion channel genes differ across nuclei, suggesting that each nucleus uses a distinct set of mechanisms to regulate features related to excitability and spike timing. Overall, the lists of differential ion channel genes in this study represent fundamental reference sets for future pharmacological and genetic manipulations.

By far the most prominent shared enrichments were for GPCR-related pathways (Table 2B). Despite the shared pathway annotation, however, closer inspection of the complements of genes underlying these GPCR-related specializations revealed they were quite different. For example, while striatal Area X had several dopaminergic receptors, pallial HVC was enriched for cholinergic receptors. These findings are consistent with the known dopaminergic and cholinergic inputs and modulations of these respective nuclei [51–53]. Thus, the specific genes identified represent well-supported candidate targets for gene and pharmacological manipulation to better understand the contribution of these neuromodulatory pathways to vocal learning.

Other more general trends included shared enrichments in pathways related to neuromodulation, neurotransmission, and peptidergic modulation, and within the nuclei of the DMP, a shared enrichment of genes in reelin-related pathways. The fact that both RELN and its VLDLR and LRP8 receptors are differential markers of DMP nuclei suggests the possible involvement of the reelin pathway in the formation of DMP connections (discussed in more detail below). The low pathway diversity in nucleus RA is also intriguing, given the large number of differential markers of this nucleus. Possible explanations include a lower diversity of cell types compared to other nuclei or a higher representation of genes per pathway, either of which would suggest that RA is highly specialized compared to other song nuclei.

### Axonal guidance and synaptic plasticity genes

The observed patterns of axonal guidance cues genes are potentially of relevance for understanding the regulation of connections across nuclei, but also raise questions about ligand-receptor pairing rules in this system. Among semaphorins and plexins, there is precedence for SEMA3E expression in presynaptic terminals having repulsive interactions with post-synaptic terminals expressing the PLXND1 receptor, but co-expression of PLXND1 with NRP1 can flip this interaction to be attractive, thus making a co-expressing nucleus an attractive target for SEMA3E-expressing terminals [54, 55]. Indeed, interactions between SEMA3E and PLXND1 play an important role in establishing specificity of cortico-thalamo-striatal connections [56]. Intriguingly, SEMA3E shows remarkably high expression in HVC and LMAN, and thus could be involved in influencing the projections of these nuclei to their common targets RA and/or striatal Area X. Consistent with this possibility, NRP1 expression is high in Area X. However, PLXND1 expression in Area X and in the striatum, as a whole was quite weak, and expression of the PLXND1/NRP1 co-receptors was not particularly marked in RA either. On the other hand, PLXND1/NRP1 are both highly expressed in HVC, which might make HVC attractive to inputs that express SEMA3E. Unfortunately, SEMA3E expression in nuclei that project to HVC (NIf, Uva, MMAN) has yet to be determined. Alternatively, there is precedence in developing sensory-motor circuits in the mammalian spinal cord for post-synaptic SEMA3E interacting with pre-synaptic PLXND1 and/or NRP [55, 56]. Under this scenario, the high expression of SEMA3E in HVC and LMAN might help to regulate inputs to these nuclei. However, this scenario seems unlikely because prominent expression of PLXND1/NRP1 appears largely lacking in nuclei that provide inputs to LMAN or HVC. In short, more work is needed to determine whether and how this particular axon guidance system operates in birds.

The high expression of PLXNA4s and NRPs in HVC and RA suggests these nuclei might be attractive targets for terminals expressing their corresponding SEMA3-type (A/C/D/F) ligands. Notably, the patterns in zebra finch are consistent with those in another vocal-learner, the Bengalese finch [57], suggesting a conserved program of axon guidance cues in the songbird lineage. While data to test this idea are not yet available, one would predict high expression of these ligands in nuclei (e.g., L/MMAN, Uva, NIf) that provide inputs to PLXNA4-expressing nuclei. SEMA7A was found to be a positive marker of all song nuclei examined, consistent with its prominent role in the maturation of mammalian cortical circuits [58]. Intriguingly, the down-regulation of the PLXNC1 receptor would make projection targets receptive to SEMA7A-positive terminals if this ligand-receptor interaction were repulsive, as in other systems [59]. Under this scenario, SEMA7A-expressing axonal terminals from a given input (this could apply to several song nuclei) would interact with PLXNC1-expressing cells outside the target nucleus and be inhibited from making contact, and/or compelled to continue towards the target by other attractants. For example, SEMA7A interacts prominently with integrin receptors, which would make the high ITGA1 expression in RA and nXIIts relevant for the HVC-to-RA and RA-to-nXIIts projections. Similarly, the high expression of PLXNC1 in DLM shell compared to its core (Fig. 14d) could play a role in the specificity of the X-to-DLM projection. The patterns observed for SEMA5A and 6D and their PLXNA4 receptors suggest similar roles in the projections of the DMP as for SEMA7A. Interestingly, the pattern of SEMA6A in zebra finch is consistent with the prominent role of this SEMA in guiding descending fibers from PLXNA4-expressing corticospinal neurons in the brainstem in mammals [60]. In this context, the high expression of PLXNA4 in RA and the prominent expression of its ligand SEMA6A in several brainstem nuclei, but down-regulation in nXIIts could help to regulate the specificity of the RA-to-nXIIts projection. In contrast to all above, the SEMA4s/PLXNBs patterns suggest a lack of prominent roles in song system projections.

Cadherins and protocadherins are a large family of genes that are involved in cell-cell adhesion and play important roles in neuronal connection formation. Our data suggests some specific song system projections where CDHs might play a role through homophilic interactions. This includes CDH6 (HVC-to-X projection), CDH13 (RA-to-nXIIts projection), and CDH9 (all projections of the DMP). The overall trend, however, was down-regulation, suggesting several CDH-related pathways are shut down in the adult zebra finch song system. Previous studies have shown differential expression of some cadherins in song nuclei of Bengalese finches [61], or explored a potential role of cadherins in vocal control [62]. While we report on a much larger set of genes in this family, consistent observations across studies include: a) the up-regulation of CDH6 (a.k.a. Cad6b) in HVC, Area X, and DM, b) up-regulation of CDH9 in HVC, c) down-regulation of CDH4 (a.k.a. Rcad) in all major forebrain song nuclei and in nXIIts, and d) down-regulation of CDH7 in RA. However, we did not observe marked up-regulation of CDH6 in RA and LMAN, nor the up-regulation of CDH10 in RA, as reported in Bengalese finches. While these could be real species differences, they could also derive from different behavioral conditions, or uncharacterized transcript variants. CDH1 was previously reported as a positive marker of HVC (Lovell et al. [24]), however a close examination of alignments to the now available zebra finch genome reveals that the reported clone is GAS8 and that CDH1 was not evaluated.

The high expression of RELN in DM and of the RELN receptors VLDLR and LRP8 in nXIIts suggests a possible role in the DM-to-nXIIts projection- an important pathway for vocal-motor patterning. Because both RELN and VLDLR are known targets of FOXP2, this brainstem vocal projection could represent a still unexplored pathway (outside of the basal ganglia) for FOXP2 modulation of vocal behaviors. Alternatively, the differential expression of RELN in DM versus nXIIts could play a role in the specificity of the projections from RA to these two brainstem targets. Our data are largely consistent with previous observations in canaries [63], although we did not detect up-regulation in Area X. Overall, the *in situ* data support the bioinformatics indication of the reelin pathway (Table 3C) as an important target of regulation, and the marked RELN down-regulation suggests suppression may be important for the maintenance of the song system circuitry. As for SLIT/ROBO genes, our data support previous observations of convergent specializations between RA and the human primary laryngeal motor cortex [23], and of differential regulation of SLITs and ROBOs in nuclei of the vocal-motor circuitry [64]. ROBOs are generally thought to be axonally-expressed receptors that have repulsive interactions with target-secreted SLIT ligands. Thus, the down-regulation of SLIT1 in RA and nXIIts could facilitate the targeting of ROBO-expressing terminals from HVC-to-RA and from RA-to-nXIIts, respectively. ROBO1 is largely down-regulated in RA but is highly expressed in a sparse cell population, which could correspond to a subset of projection neurons. Alternatively, the SLIT down-regulation in HVC and RA, coupled with the up-regulation of ROBOs in RA and nXIIts, might play a role in the targeting specificity of the HVC-to-RA and the RA-to-nXIIts projections. This notion has been previously suggested as helping to establish the specificity of the RA-to-nXIIts projection [64], a connection critical for learned vocalizations, but it would require the pre- and post-synaptic localizations of SLITs and ROBOs, respectively. The lack of prominent expression of SLITs or ROBOs in DM further supports a differential role of these genes in the RA-to-nXIIts projection. Lastly, the prominent differential regulation within these gene families in AFP nuclei (Area X, DLM, LMAN) suggests further roles in other parts of the song system.

The marked differential regulation of UNC5s in all song nuclei, including RA and its targets DM and nXIIts (Fig. 6c), suggests a prominent role for this pathway in song circuit connectivity. Interestingly, axonal terminals that express UNC5 receptors are repulsed by their main known ligands (netrins; [65, 66]). Our limited data suggest a lack of prominent regulation of netrins in the forebrain, but expression analysis of other netrins and examination of brainstem nuclei would be important next steps to establish the role of UNC5s. We also note that FLRTs, which provide alternative non-netrin repulsive cues to UNC5 receptors [67], and NEO1 (a.k.a. DCC), which has been implicated in chemo-attraction of netrins [68], are respectively up- and down-regulated in HVC and its targets, further suggesting UNC5-related effects at multiple sites in the song circuitry. Other classes of guidance cues (NTNGs-, NLGNs- and RTN4-related) showed down-regulation trends, with no apparent coordinated expression of known ligand-receptor pairs between connected song nuclei. We did not observe an enrichment of NTNGs in thalamo-cortical projection neurons (e.g. DLM) as reported in mammals [50, 69]. Lastly, the general down-regulation of No-Go-related cues and receptors (LINGOS, RTN4s) suggests that suppression of this axonal guidance pathway is a prominent feature of the mature song circuitry.

The marked differential expression of axonal guidance and connectivity cue genes in adult song nuclei suggests that the maintenance and/or plasticity of song system projections is under active regulation in adults. In several cases we did not see coordinated regulation of known ligand-receptor pairs in connected nuclei, suggesting that not all ligand-receptor interactions in songbirds conform to known rules of axon guidance. We note that most such rules derive from work in non-avian organisms that do not possess brain pathways for learned vocalizations. Thus, the patterns observed in finches might reflect axon guidance rules that are unique to birds, or related to the emergence of the song system in songbirds. A deeper understanding of how axonal guidance cues modulate song system connections requires developmental studies, a definition of the cellular distribution of proteins in pre-synaptic vs. post-synaptic structures, and gene manipulations to test specific hypotheses.

### Excitatory and inhibitory neurotransmitter receptors

Significant insights were also gained from the analysis of inhibitory and excitatory neurotransmitter receptors. Consistent with their general roles, GABAergic and glutamatergic receptor families were broadly expressed in all song nuclei, but several genes showed evidence of regulated expression. For GABAergic receptors, the non-differential expression of the more abundant and fundamental alpha subunits of type-A receptors contrasted markedly with the differential regulation of multiple beta, delta, and epsilon subunits, suggesting these less prevalent subunits are more prominent targets of differential regulation in the song system. In particular, the up-regulation of delta subunits in all major telencephalic song nuclei, consistent with a previous report [70], suggests a distinctive feature of inhibitory neurotransmission, but its functional significance is unknown. The data on glycine receptor subunits is also intriguing, as the patterns of alpha and beta subunits seem largely complementary rather than overlapping. These receptors play important roles during development, and in adults tend to be more constrained to brainstem regions, including auditory nuclei [71–74]. Since functional receptors depend on the presence of both alpha and beta subunits, the patterns in adult finches suggest that they may play other roles, rather than reflecting functional glycine receptors.

The glutamate receptor patterns were largely consistent with previous data [26], both studies pointing to the differential regulation of multiple subunits at multiple sites as a distinctive feature of the song system. The possible implications of the observed patterns for regulating firing, deactivation, and plasticity within song nuclei (discussed more extensively in Wada *et al*. [26]), are not clear in the absence of pharmacological or genetic manipulations. While the present study presents the first expression data on delta-subunits in the song system, other differences between studies include the differential expression of GRIA3 in HVC and GRM4 in LMAN in the present study only, and of GRIK2/GRM8 in LMAN, GRM1/GRM5 in Area X, and GRM8 in HVC, seen by Wada *et al*. only. We also note that gene down- or up-regulation in distinct cell populations is more readily apparent with the non-radioactive approach used here (e.g. Fig. 12h, GRID1 in Area X; Figs. 10k-m, GRM1, GRID1, and GRID3 in HVC). Moreover, there is evidence of alternative splicing among zebra finch glutamate receptors subunits (e.g. Wada *et al.* [26], Fig. 5), which could further contribute to observed differences between the studies.

The expression of neuromodulatory receptors was consistent with and extends previous studies, and may help explain the differential ligand binding in autoradiographic assays. For example, the high expression of ADRA2A in HVC, LMAN, and to a lesser extent Area X, provides a likely basis for the high binding of alpha-2 adrenergic ligands to these nuclei [75]. However, the high alpha-2 ligand binding to RA and the modulation of RA via alpha-2 agonists/antagonists [76] remain unexplained, as ADRA2A was non-differential and ADRA2C was down-regulated in RA. The data on dopaminergic receptors are consistent with the more extensive study by [77], but the down-regulation of DRD2 in RA and up-regulation of DRD5 in HVC were not previously seen. With regards to DRD7, a gene markedly differential in several song nuclei, we adopted a term that better reflects its presence throughout the vertebrate phylogeny (DRD7 in *Danio rerio*, previously D1DR in finch [77] and DD1CR in chicken) and absence in mammals. As for serotonergic receptors, the marked up-regulation of multiple subunits in forebrain nuclei (except for HTR1B down-regulation HVC) points to serotonergic modulation as a major feature of the song system, consistent with pharmacological and electrophysiological studies (e.g. [78]). For cholinergic receptors, the data confirm the high expression of CHRM4 in Area X previously seen by Lovell et al. [24], and provide a basis for the higher binding of a muscarinic cholinergic antagonist to Area X compared to the surrounding striatum [79]. In contrast, the low expression of CHRM5 in RA might contribute to the low ligand binding described for this nucleus. We have also confirmed the high expression of CHRNA5 and CHRNA7 in HVC (previously seen by Lovell et al. [24]). This is consistent with the high alpha bungarotoxin binding for these nicotinic receptors in HVC [80]. *In situ* hybridization shows that CHRN4 is highly expressed in a sparse cell population within Area X. Of note, genomic alignments show that the cDNA clone annotated as CHRNA2 in Lovell et al. [24] actually corresponds to CHRNA4, thus the CHRNA2 expression pattern remains unknown in zebra finch. Overall, the data point to HVC and Area X as major targets of modulation by cholinergic inputs, particularly via CHRNA7 receptors, consistent with previous pharmacological and physiological data [81].

Our data are also consistent with and expand upon previous reports of peptide-related regulation in the song system based on gene expression and immunostaining data [24, 82–84]. In fact, peptide-related patterns provide some striking examples of differential regulation, suggesting important contributions to the physiology of the song system. They also often occur in sparse cell populations, suggesting roles in neuronal cell type diversification. We note that differential SST expression was not detected in HVC or RA, which contrasts with the described immunostained somata in these nuclei in comparison with the adjacent tissues [83]. Further, VIP was down in RA and Area X, consistent with [85], but PENK (MET) was not up in RA as expected based on the peptide distribution, suggesting that differential translation plays a role in this differential regulation. TAC1 is a remarkable marker of Area X (also reported in [86]), but is also highly expressed in nucleus accumbens (www.zebrafinchatlas.org). This finding likely explains why TAC1 was not detected as differential in Area X on the array. We also note that expression of opioid receptors in song nuclei was not detectable with our *in situ* protocol. This differs from reports suggesting that mu-opioid mRNA expression is not only detected, but marginally higher in HVC and RA than in surrounds [87]. We note, however that this previous report contradicts previous ligand binding autoradiographic studies in another songbird (Dark-eyed Junco) that reported no apparent differences in binding of mu-opioid ligands in the song system [88].

## Conclusions

Our study provides the most comprehensive characterization to date of transcriptional and molecular specializations of the zebra finch song system. We have identified over 3300 genes that constitute specializations of one or more vocal nuclei. Pathway enrichment analysis provides novel insights into the molecular constituents that underlie specific functional properties of individual nuclei. In general, the data point to G-protein receptor activation, axon guidance, and the regulation of membrane potential by voltage-gated K+ channels as being critical specializations of the song system. More specifically, we also discovered very specific markers of each nucleus that have strong links to the establishment and maintenance of neuronal projections, the regulation of neuronal excitability, and the modulation of neurotransmission and synaptic function. Finally, curated lists of genes provide a wealth of potential new targets for pharmacological and gene manipulation, and will serve as an important resource for building and testing hypotheses about the roles that individual genes play in defining properties such as cellular excitability, neurotransmission and neuromodulation, and circuit connectivity.

## Additional files

Additional file 1: **Table S1.** Summary of the numbers of oligos and cDNAs analyzed for each nucleus. **Table S2.** Summary of the subsets of differential vs. non-differential genes analyzed for each nucleus to establish the *p*-value cutoffs in Fig. 2. **Table S3.** List of genes that are differentially regulated in HVC vs. Shelf. **Table S4.** List of genes that are differentially regulated in RA vs. VLA. **Table S5.** List of genes that are differentially regulated in nXIIts vs. SSP. **Table S6.** List of genes that are differentially regulated in Area X vs. VSP. **Table S7.** Pathways significantly over-represented in HVC. **Table S8.** Pathways significantly over-represented in RA. **Table S9.** Pathways significantly over-represented in nXIIts. **Table S10.** Pathways significantly over-represented in Area X. **Table S11.** Level 5 Gene Ontology (GO) terms significantly over-represented in HVC. **Table S12.** Level 5 Gene Ontology (GO) terms significantly over-represented in RA. **Table S13.** Level 5 Gene Ontology (GO) terms significantly over-represented in nXIIts. **Table S14.** Level 5 Gene Ontology (GO) terms significantly over-represented in Area X. **Table S15.** Unique and shared markers of song system nuclei. **Table S16.** Summary of in situ and array data analyzed to assess gene regulation in Figs. 4-9. (XLSX 244 kb)

Additional file 2: **Figure S1.** Identification and removal of oligos with low normalized intensity values. For each microarray screening experiment (A: RA vs. VLA; B: nXIIts vs. SSP; C: Area X vs. VSP), we ranked each set of oligos from high-to-low according to normalized average expression values measured for each sample type, in each pair of samples (n=3 samples per region). An average signal intensity versus an oligo’s ranking was then plotted and the resulting curves (in blue) were examined to determine whether there was an obvious inflection or shoulder (indicated by arrowheads in the inset graphs in A-C) in the distribution indicating a lack of detectability in the signal. This inflection point was used to establish minimum signal detection cut-off limits for each experiment. Oligos with an average maximum intensity value less than the average value at the inflection point plus 2.5 times the standard deviation of that value were removed from further analysis. **Figure S2.** Assessment of gene regulation via UCSC’s ‘Genome Browser’ and custom BED-tracks. To more accurately assess the occurrence and/or direction of differential regulation for the genes presented in Figs. 4, 5, 6, 7, 8, 9 and 10, we examined each gene’s locus in the genome (e.g. OPRL1) and assessed the regulation of oligos derived from each experiment based on their association with the locus. OPRL1 (on chr 20) is predicted to consist of three exons (‘Ensembl Gene Predictions - 89’ track; transcript model ENSTGUT00000007601.1 in red). Alignments of Refseqs from various species (‘Non-Zebra Finch RefSeq Genes’ track in blue) extend this gene model to include both 3’ and 5’-untranslated regions of OPRL1. For each experiment we constructed a custom BED-track consisting of an oligo’s genomic position, and its regulation (+, constructed a custom BED-track consisting of an oligo’s genomic position, and its regulation (+, -, non-differential) in each nucleus based on the cut-off values established in Fig. 2. This track was uploaded as a custom track to the UCSC’s browser (alignments shown under ‘SS Marker By Gene’ track). Individual cDNAs/oligos are color coded according to their assessed regulation in each experiment (i.e. green = up-regulated; red = down-regulated; black = assessed, but not differentially regulated), and each oligo is identified by a unique identifier (i.e. oligo/cDNA ID_nucleus_regulation). In the example shown, OPRL1 is associated with multiple oligos that were assessed variously in the array experiments. The regulation of OPRL1 in each nucleus is determined by applying a majority rule scoring assessment based on counts of unique significant (shaded in red or green) and non-significant (shaded in black) oligos. Accordingly, OPRL1 is up-regulated in nXIIts, down-regulated in RA and Area X, and non-differential in HVC. Of note, this approach helps to identify cases where nearly identical (i.e. non-unique) oligos are assessing the expression of the same gene in a given structure (e.g. 0064P0001D08 and 0063P0014E01 in RA, nXIIts, and Area X), and thus cannot be considered independent assessments of that gene. This approach also identifies cases where nearly identical oligos give conflicting information regards to regulation (e.g. 0063P0014E01 with 0064P0001D08 in RA), as well as non-identical oligos probing the same exon that are giving conflicting results (0063P0014E01 conflicts with 0203P0048K03 and 0106P0001G10 for RA). These issues are addressed by collapsing non-unique oligos and repeating the majority rule scoring. (PPTX 188 kb)
