# Supplementary figures and images for: The constitutive differential transcriptome of a brain circuit for vocal learning
Source: BMC Genomics. 2018 Apr 3;19:231. doi: 10.1186/s12864-018-4578-0 (PMC5883274; doi:10.1186/s12864-018-4578-0)

## Slide 1
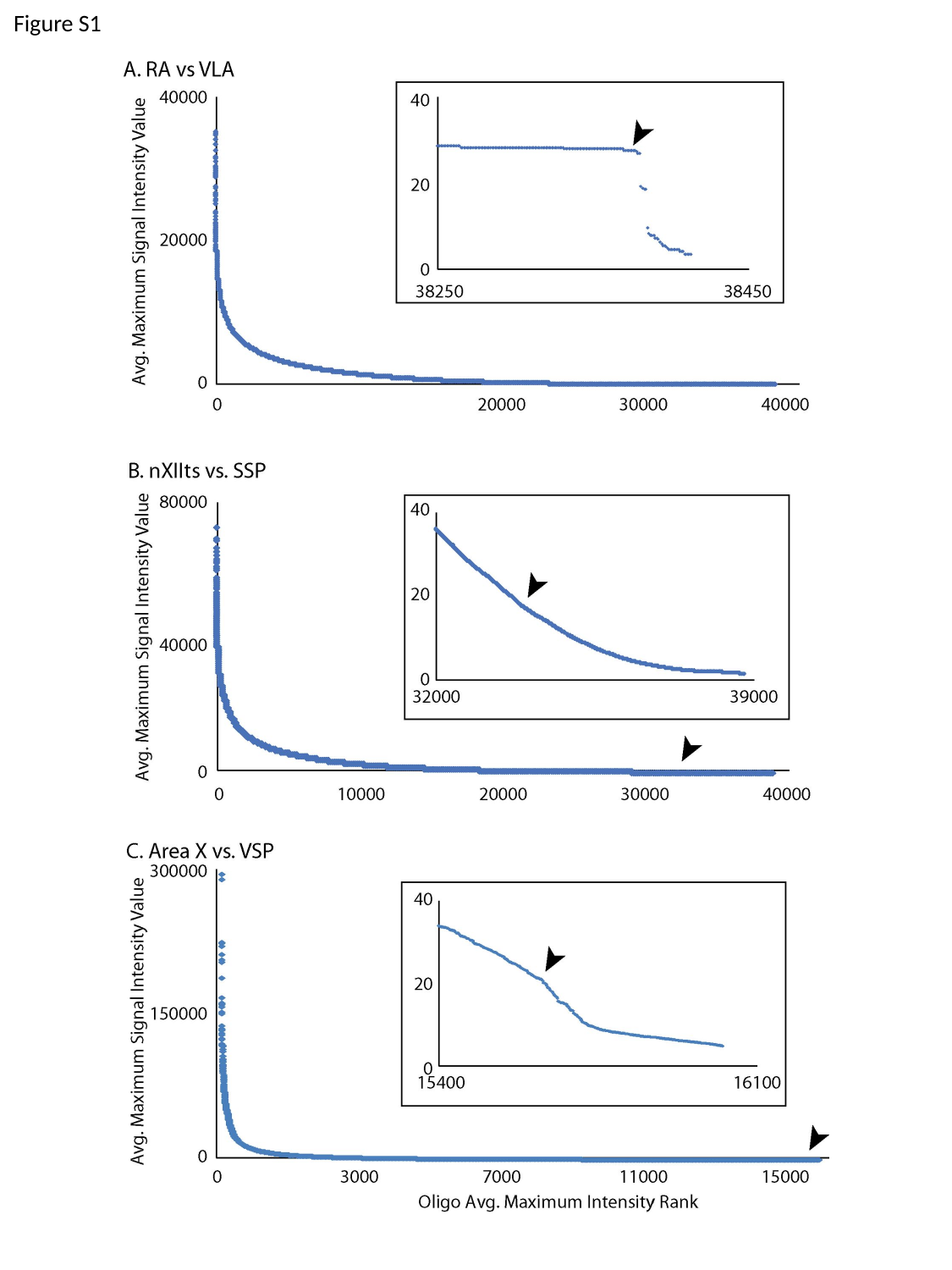

Figure S1

## Slide 2
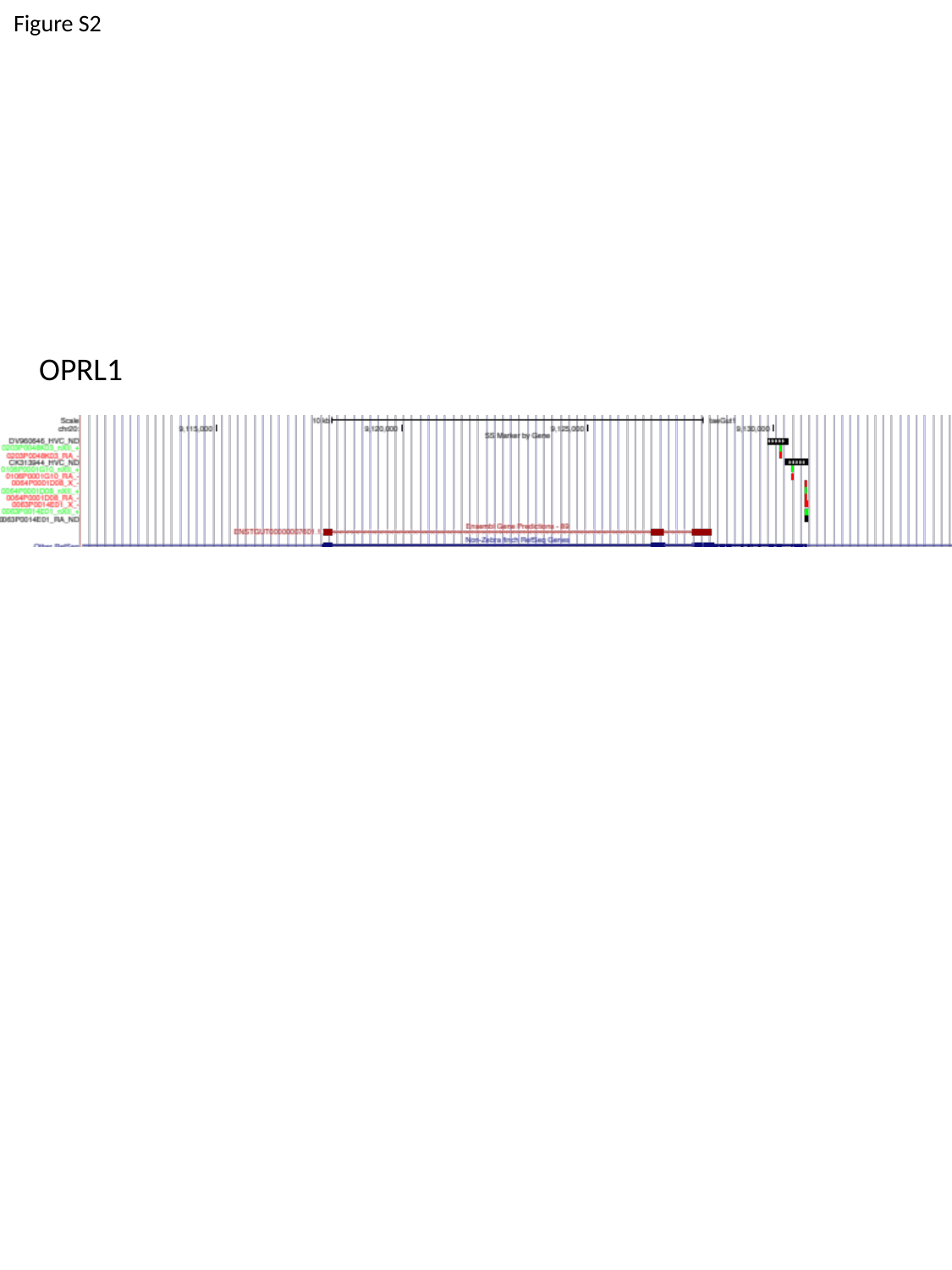

Figure S2
OPRL1

Supplement: Supplementary file 2 — Figure S1. Identification and removal of oligos with low normalized intensity values. For each microarray screening experiment (A: RA vs. VLA; B: nXIIts vs. SSP; C: Area X vs. VSP), we ranked each set of oligos from high-to-low according to normalized average expression values measured for each sample type, in each pair of samples (n=3 samples per region). An average signal intensity versus an oligo’s ranking was then plotted and the resulting curves (in blue) were examined to determine whether there was an obvious inflection or shoulder (indicated by arrowheads in the inset graphs in A-C) in the distribution indicating a lack of detectability in the signal. This inflection point was used to establish minimum signal detection cut-off limits for each experiment. Oligos with an average maximum intensity value less than the average value at the inflection point plus 2.5 times the standard deviation of that value were removed from further analysis. Figure S2. Assessment of gene regulation via UCSC’s ‘Genome Browser’ and custom BED-tracks. To more accurately assess the occurrence and/or direction of differential regulation for the genes presented in Figs. 4, 5, 6, 7, 8, 9 and 10, we examined each gene’s locus in the genome (e.g. OPRL1) and assessed the regulation of oligos derived from each experiment based on their association with the locus. OPRL1 (on chr 20) is predicted to consist of three exons (‘Ensembl Gene Predictions - 89’ track; transcript model ENSTGUT00000007601.1 in red). Alignments of Refseqs from various species (‘Non-Zebra Finch RefSeq Genes’ track in blue) extend this gene model to include both 3’ and 5’-untranslated regions of OPRL1. For each experiment we constructed a custom BED-track consisting of an oligo’s genomic position, and its regulation (+, constructed a custom BED-track consisting of an oligo’s genomic position, and its regulation (+, -, non-differential) in each nucleus based on the cut-off values established in Fig. 2. This track was uploa [file 12864_2018_4578_MOESM2_ESM.pptx]
